# Intronic *Cis*-Regulatory Modules Mediate Tissue-Specific and Microbial Control of *angptl4*/*fiaf* Transcription

**DOI:** 10.1371/journal.pgen.1002585

**Published:** 2012-03-29

**Authors:** J. Gray Camp, Amelia L. Jazwa, Chad M. Trent, John F. Rawls

**Affiliations:** 1 Department of Cell and Molecular Physiology, The University of North Carolina at Chapel Hill, Chapel Hill, North Carolina, United States of America; 2 Department of Microbiology and Immunology, The University of North Carolina at Chapel Hill, Chapel Hill, North Carolina, United States of America; Johns Hopkins University, United States of America

## Abstract

The intestinal microbiota enhances dietary energy harvest leading to increased fat storage in adipose tissues. This effect is caused in part by the microbial suppression of intestinal epithelial expression of a circulating inhibitor of lipoprotein lipase called Angiopoietin-like 4 (Angptl4/Fiaf). To define the *cis*-regulatory mechanisms underlying intestine-specific and microbial control of *Angptl4* transcription, we utilized the zebrafish system in which host regulatory DNA can be rapidly analyzed in a live, transparent, and gnotobiotic vertebrate. We found that zebrafish *angptl4* is transcribed in multiple tissues including the liver, pancreatic islet, and intestinal epithelium, which is similar to its mammalian homologs. Zebrafish *angptl4* is also specifically suppressed in the intestinal epithelium upon colonization with a microbiota. *In vivo* transgenic reporter assays identified discrete tissue-specific regulatory modules within *angptl4* intron 3 sufficient to drive expression in the liver, pancreatic islet β-cells, or intestinal enterocytes. Comparative sequence analyses and heterologous functional assays of *angptl4* intron 3 sequences from 12 teleost fish species revealed differential evolution of the islet and intestinal regulatory modules. High-resolution functional mapping and site-directed mutagenesis defined the minimal set of regulatory sequences required for intestinal activity. Strikingly, the microbiota suppressed the transcriptional activity of the intestine-specific regulatory module similar to the endogenous *angptl4* gene. These results suggest that the microbiota might regulate host intestinal Angptl4 protein expression and peripheral fat storage by suppressing the activity of an intestine-specific transcriptional enhancer. This study provides a useful paradigm for understanding how microbial signals interact with tissue-specific regulatory networks to control the activity and evolution of host gene transcription.

## Author Summary

Recent studies have revealed that the community of microorganisms residing in the intestine regulates fat storage. Microbes evoke this response in part by suppressing expression of the *Angptl4* gene, which encodes a secreted inhibitor of fat storage. Although *Angptl4* is expressed in multiple tissues, microbial suppression occurs only in the intestine. To determine how microbes control fat storage, we must elucidate the mechanisms underlying intestine-specific and microbial regulation of *Angptl4* expression. Here, we take advantage of the unique features of the zebrafish model to define the regulatory DNA sequences controlling *angptl4* expression. Our results reveal that different DNA regulatory regions within the *angptl4* gene mediate expression of *angptl4* in the intestine and other tissues. By assessing the evolution of *angptl4* regulatory regions and subjecting them to structure-function analyses, we identify discrete DNA sequences that are required for intestinal expression. Strikingly, microbes suppress the activity of the intestine-specific regulatory region similar to the endogenous *angptl4* gene. Therefore, intestinal microbes might regulate *angptl4* production by suppressing the signaling pathway interpreted by an intestine-specific transcriptional regulatory region. Our results provide new mechanistic insights into how intestinal microbes might influence fat storage and contribute to the development of obesity.

## Introduction

The vertebrate intestine harbors a dense community of microorganisms (gut microbiota) that exerts a profound influence on distinct aspects of host physiology [1], [2]. The gut microbiota has been identified as a potent environmental factor in a growing number of human diseases, including inflammatory bowel disease [3], antibiotic-associated diarrheas [4], cardiovascular disease [4], and obesity [5]. As a consequence, there is considerable interest in understanding the mechanisms by which this resident microbial community influences health and disease in humans and other animals.

The ability of the microbiota to modify host nutrient metabolism and energy balance is a prominent theme in host-microbe commensalism in the intestine. Recent mechanistic insights into this process have been provided by comparisons between mice reared in the absence of microbes (germ-free or GF) to those colonized with members of the normal microbiota, as well as high-throughput DNA sequencing analysis of the metabolic potential of gut microbial genomes. These approaches have shown that the gut microbiota contributes biochemical activities not encoded in the host genome that enhance digestion of dietary nutrients [6], [7]. The resulting increase in digestive efficiency results in elevated plasma levels of triglyceride (TG)-rich lipoproteins [8], [9]. TG within circulating lipoprotein particles is hydrolyzed through the rate-limiting activity of lipoprotein lipase (LPL) located at the luminal surface of capillaries. TG hydrolysis releases free fatty acids (FFA) for uptake by adjacent tissues for oxidation (e.g., in cardiac and skeletal muscle) or fat storage (e.g., in adipose tissues) [10]. The presence of a gut microbiota also results in a concomitant reduction in intestinal expression of *Angiopoietin-like 4* (*Angptl4*, also called *Fiaf*, *Pgar*, and *Hfarp*) [8], [11], encoding a circulating peptide hormone that acts as a direct inhibitor of LPL activity [12]–[15]. Studies in gnotobiotic mice have indicated that microbial suppression of *Angptl4* expression is restricted to the intestinal epithelium and is not observed in other tissues that express *Angptl4*, such as liver and adipose tissue. This restricted suppression leads to a significant increase in LPL activity and fat storage in adipose tissue of animals colonized with a microbiota, which is an effect abolished in mice lacking *Angptl4* [8]. These results have established *Angptl4* as a key host factor mediating the microbial regulation of host energy balance and have raised considerable interest in defining the mechanisms underlying the tissue-specific and microbial regulation of *Angptl4* expression. The importance of understanding mechanisms regulating Angptl4 production is further underscored by reports suggesting that human ANGPTL4 functions as an important determinant of plasma TG levels [16], [17] and by Angptl4's additional functions in angiogenesis [18], tumor cell survival [19] and metastasis [20], [21], and wound healing [22].

Previous studies have revealed that mammalian *Angptl4* expression is subject to complex cell type-specific regulation but the underlying mechanisms remain unclear. *Angptl4* mRNA in humans and rodents is expressed in multiple tissues, including adipose tissue, liver, intestinal epithelium, pancreatic islets, and cardiac and skeletal muscle [8], [19], [23]–[26]. Preliminary insights into the *trans*- and *cis*-regulatory mechanisms controlling *Angptl4* transcription have been provided by analyses in non-intestinal tissues. Members of the peroxisome proliferator-activated receptor (PPAR) family of nuclear receptors (i.e., PPARγ, PPARα, and PPARβ/δ) have been identified as activators of *Angptl4* expression in adipose tissue, liver [23], [27], skeletal [28] and cardiac muscle [29], myofibroblasts [30], and colon carcinoma cells [31]. A PPAR-responsive element (element defined as a transcription factor binding site or TFBS) located in the proximal portion of *Angptl4* intron 3 has been shown to directly bind different PPAR family members in adipose tissue, liver [27], and myofibroblasts [30]. Additional studies in non-intestinal cell types have identified functional TFBSs for SMAD3 and glucocorticoid receptor in the 5′ distal region and 3′ untranslated region (UTR), respectively [30], [32]. *Angptl4* transcription is induced under hypoxic conditions in several non-intestinal cell types by hypoxia-inducible factor 1α (HIF1α) [33], [34]; however, the TFBSs mediating this response have not been identified. These studies support a role for these *trans*- and *cis*-regulatory factors in controlling *Angptl4* transcription in these cell types, yet the mechanisms underlying the transcription of *Angptl4* in other tissues, such as the intestine and pancreatic islet, remain unknown. Moreover, the *cis*/*trans*-regulatory mechanisms underlying microbial suppression of *Angptl4* transcription in the intestinal epithelium remain undefined.

The zebrafish (*Danio rerio*) provides unique opportunities to study the transcriptional regulatory programs mediating tissue-specific and the microbial control of vertebrate gene expression. Robust transgenesis methods using the Tol2 transposon system [35], large numbers of offspring, and optical transparency facilitate efficient spatiotemporal analysis of reporters driven by potential DNA regulatory regions in mosaic and stable transgenic animals [36]. The anatomy and physiology of the zebrafish digestive tract are highly similar to mammals, including an intestine, liver, gall bladder, and exocrine and endocrine pancreas [37]–[39]. The intestinal epithelium of the zebrafish displays proximal-distal functional specification and is composed of absorptive enterocytes as well as secretory goblet and enteroendocrine lineages [40], [41]. The zebrafish intestine is colonized by a microbiota shortly after the animals hatch from their protective chorions at 3 days post-fertilization (dpf) [42], [43] and reaches a stage sufficient to support nutrient digestion by 5 dpf [44]. To study the roles of commensal microbes on zebrafish development and physiology, we have developed methods for rearing GF zebrafish and colonizing them with members of the normal zebrafish microbiota [45], [46]. By combining these methods with functional genomic approaches, we identified zebrafish transcripts that display altered expression levels in animals raised GF compared to those colonized with a normal microbiota, including microbial suppression of a zebrafish homolog of mammalian *Angptl4* [47]–[49]. The expression pattern of this zebrafish *Angptl4* homolog, and the mechanisms underlying the tissue-specific and microbial regulation of its expression, have not been previously described.

These features position the zebrafish as a powerful model for assaying the regulatory potential of DNA involved in mediating cell-specific and microbe-responsive transcriptional events. Previous studies of DNA regulatory potential in the zebrafish system have focused primarily on developmental genes [50]–[54], and it remains unclear if the lessons learned from these analyses [55] will apply to physiologic genes like *Angptl4* that are regulated by endogenous as well as exogenous cues. Moreover, a paucity of available genome sequences for teleost species closely related to zebrafish has severely limited prior evolutionary analysis of *cis*-regulatory sequence and function. Here, we utilize the zebrafish to investigate the *cis*-regulatory mechanisms governing tissue-specific and microbial control of *Angptl4* transcription. We focus our analysis on intestinal and islet expression, where the mechanisms regulating *Angptl4* transcription have not been adequately examined. We first uncover distinct intronic *cis*-regulatory modules (CRM, defined here as a discrete DNA region containing sufficient information to confer a regulatory function) that mediate intestinal and islet expression. Using this information, we reveal that the intestine-specific CRM also responds to microbial stimuli to suppress *angptl4* expression. These results provide novel insights into how vertebrates might control the tissue-specific transcription of *Angptl4* and constitute an important advance towards understanding how commensal gut microbes regulate gene expression and energy balance in their vertebrate hosts.

## Results

### Tissue-specific expression of zebrafish *angptl4*

**Figure 1:**
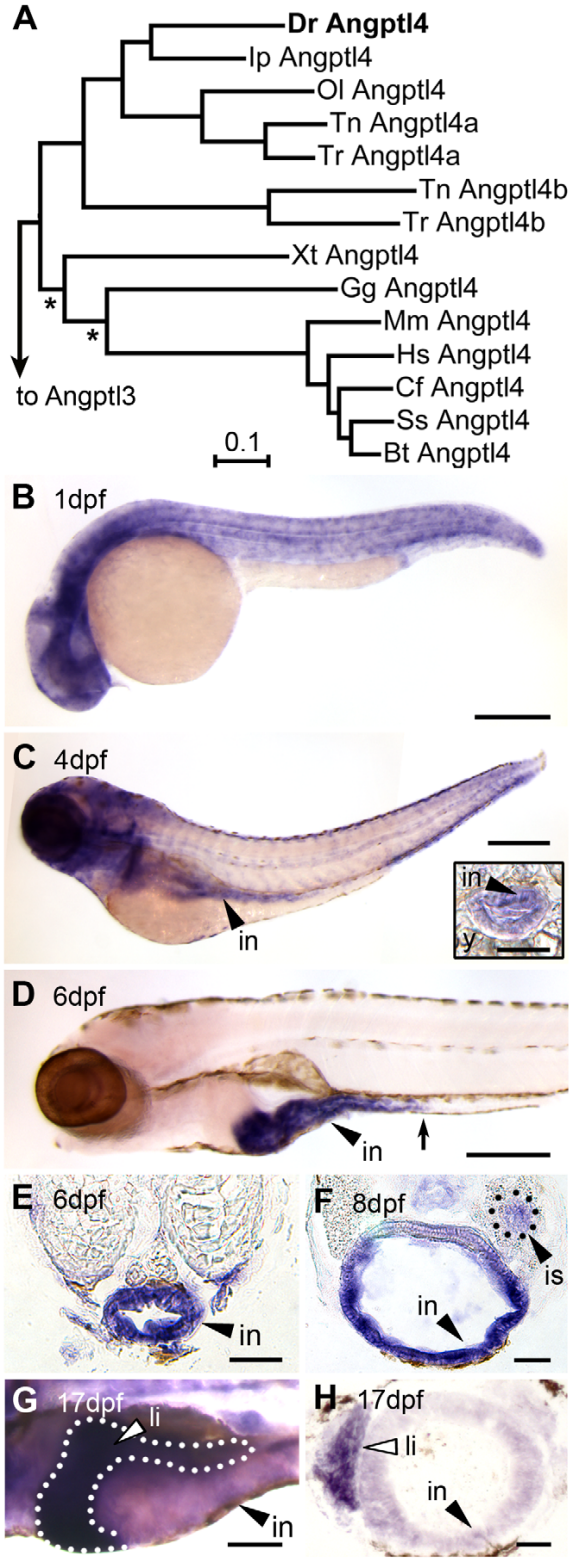
Tissue-specific expression of zebrafish *angptl4* mRNA. (A) Distance phylogram of Angptl4 protein from zebrafish (*Dr*, *Danio rerio*), catfish (*Ip*, *Ictalurus punctatus*), medaka (*Ol*, *Oryzias latipes*), tetraodon (*Tn*, *Tetraodoan nigroviridis*), fugu (*Tr*, *Takifugu rubipres*), xenopus (*Xt*, *Xenopus tropicalis*), chicken (*Gg*, *Gallus gallus*), mouse (*Mm*, Mus *musculus*), human (*Hs*, *Homo sapiens*), dog (*Cf*, *Canis familiaris*), pig (*Ss*, *Sus scrofa*), cow (*Bt*, *Bos taurus*). All nodes are significant (>700/1000 bootstrap replicates) except those marked with an asterisk (*). Scale bar indicates phylogenetic distance, in number of amino acid substitutions per site. We found that the genomes of zebrafish, channel catfish (*Ictaluris punctatus*), and medaka (*Oryzias latipes*) encode a single ortholog of mammalian Angptl4, whereas two pufferfish species (*Takifugu rubripes* and *Tetraodon nigroviridis*) encode two Angptl4 paralogs. See also Figure S1. (B–G) Whole-mount *in situ* hybridization (WISH) using a riboprobe targeting *angptl4* mRNA during various stages in zebrafish development reveals dynamic spatiotemporal gene expression patterns. (B) At 1 day post fertilization (dpf) embryos exhibit ubiquitous expression of *angptl4*. (C–D) By 4 dpf, marked expression is observed in the intestinal epithelium (in, black arrowhead), but by 6 dpf, robust expression becomes largely localized to the intestine (black arrowhead) and pancreatic islet (not shown). The black arrow marks the boundary between the anterior intestine (segment 1) and mid-intestine (segment 2). Scale bars = 500 µm. (E–F) Transverse sections of 6 dpf and 8 dpf animals confirm expression in the intestinal epithelium (E, in, black arrowhead) and pancreatic islet (F, is, black triangle). Scale bars = 50 µm. (G–H) At 17 dpf, strong expression is observed in the liver (li, white arrowhead, dotted line outlines the liver). G, Scale bar = 250 µm; H, Scale bar = 50 µm.

A comparative sequence analysis revealed that the zebrafish genome encodes a single ortholog of mammalian Angptl4 that displays marked amino acid sequence conservation with other vertebrate homologs (See Text S1, Figure 1A, Figures S1 and S2). We used RNA whole-mount *in situ* hybridization (WISH) to identify the tissues in which *angptl4* is transcribed during zebrafish development. We found that zebrafish *angptl4* mRNA is expressed ubiquitously in 1 dpf embryos (Figure 1B) but becomes enriched in specific tissues during post-embryonic stages. Transcripts for *angptl4* are enriched in the intestinal epithelium by 4 dpf, shortly after the intestinal tract becomes completely patent (Figure 1C), and become localized to the anterior intestine (segment 1) by 6 dpf (Figure 1D, 1E). Transcripts for *angptl4* were also enriched in the pancreatic islet by 8 dpf (Figure 1F) and in the liver by 17 dpf (Figure 1G, 1H). Notably, the intestinal epithelium [8], [11], liver [24], [27], and pancreatic islet [25] in mammals also express *Angptl4* mRNA. These data establish that the zebrafish *angptl4* ortholog is expressed in a tissue-specific pattern that is conserved across vertebrate lineages and suggest that the underlying transcriptional regulatory mechanisms may also be conserved.

### Conservation in DNA sequence guides *cis*-regulatory module discovery

**Figure 2:**
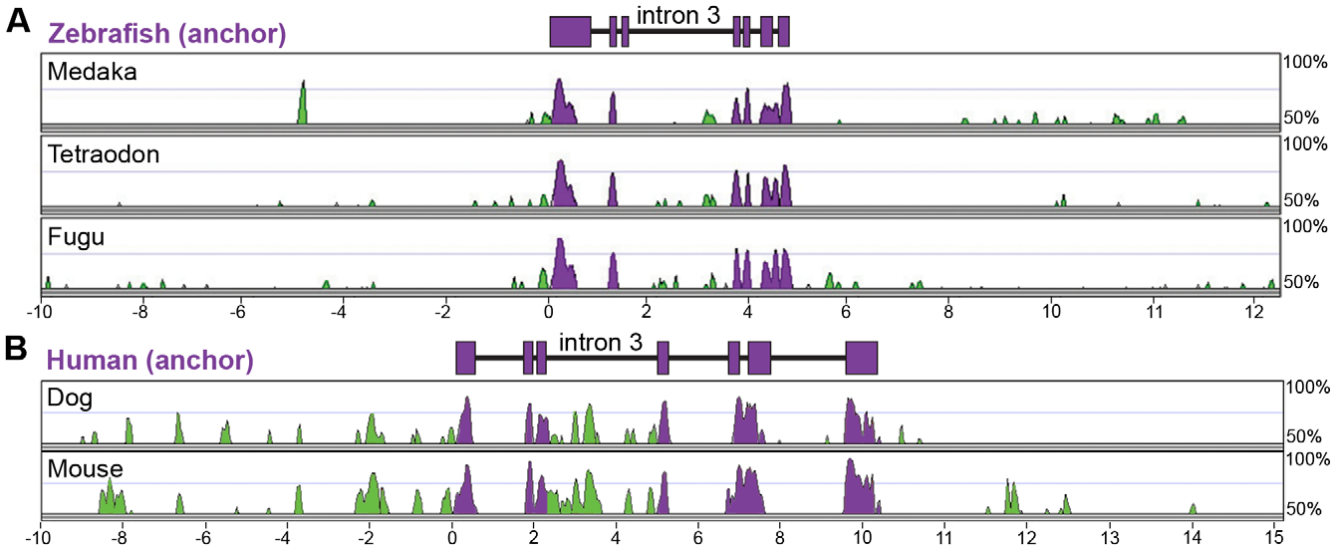
Multiple-species alignments reveal conservation in *angptl4* gene structure and location of conserved non-coding regions. (A) VISTA plot displaying the global pairwise alignment of the zebrafish *angptl4* locus with the orthologous medaka, tetraodon, and fugu regions and (B) human *ANGPTL4* locus with the orthologous mouse and dog regions. Purple conservation peaks correspond to exonic sequences, and green conservation peaks represent non-coding sequences. The zebrafish and human gene structure are denoted by purple boxes above the corresponding VISTA plot (VISTA parameters: 100 bp sliding window, LAGAN alignment). Note that the concentration of conservation peaks within intron 3 of both teleost and mammalian *angptl4* genes.

Previous studies have indicated that conservation in non-coding genomic DNA sequence across vertebrate lineages can be a reliable predictor of *cis*-regulatory DNA regions [56], [57]. We therefore used this approach to discover regulatory regions controlling transcription of *angptl4* in the liver, islet, and intestinal epithelium. Mammals and teleost fishes diverged approximately 438–476 million years ago [58], whereas zebrafish (clade Otocephala) diverged from other teleost fishes with currently-available genome sequence [clade Euteleostei; i.e., medaka (*Oryzias latipes*), stickleback (*Gasterosteus aculeatus*), fugu (*Takifugu rubripes*), and tetraodon (*Tetraodon nigroviridis*)] approximately 230–307 million years ago [59]. We generated multiple-species LAGAN alignments with Vista software using 10 kb of genomic sequence surrounding and including the *angptl4* loci from four teleost fishes (zebrafish, medaka, tetraodon, fugu) and three mammals [human (*Homo sapiens*), dog (*Canis familiaris*), and mouse (*Mus musculus*)]. Alignment of teleost and mammalian genomic sequences did not detect regions of primary sequence conservation within *angptl4* non-coding regions (>50% over 100 bp; data not shown), suggesting that these alignment methods are not sufficiently sensitive to detect existing non-coding conservation [56] or that the composition and/or location of non-coding regulatory regions are not stringently conserved between these lineages. We therefore separately aligned teleost *angptl4* (Figure 2A) and mammalian *Angptl4* loci (Figure 2B) and searched for non-coding sequence conservation in each lineage. These alignments revealed that human and zebrafish *angptl4* loci both contain 7 conserved exons as well as a concentration of conserved non-coding sequences directly upstream of exon 1 and in intron 3 (Figure 2). Similarities in gene structure and locations of conserved non-coding regions, in addition to conservation in gene expression patterns, support the hypothesis that the regulatory mechanisms of *angptl4* transcription may be evolutionarily conserved.

### The *angptl4* proximal promoter does not recapitulate mRNA expression patterns

We assayed the regulatory potential of DNA upstream and proximal to the zebrafish *angptl4* transcription start site (TSS) for the ability to transcribe a reporter in the intestine, liver, and islet. We first employed 5′ rapid amplification of cDNA ends (5′RACE) to determine the location of the TSS (Figure S3B). We identified a single TSS located 89 base pairs (bp) upstream of the translation start site and a canonical TATA box at position −31 bp of the TSS (Figure S3B). Based on this analysis and expressed sequence tag (EST) coverage of the zebrafish *angptl4* locus (data not shown), we found no evidence of alternative promoters farther upstream of the defined TSS. Using Tol2 transposon transgenesis, we assayed the regulatory potential of genomic DNA upstream of the zebrafish *angptl4* TSS, including the 5′ untranslated region (UTR) (Figure S3A), to drive expression of an enhanced green fluorescent protein (GFP) reporter in 0–7 dpf zebrafish larvae. We found that regulatory DNA within −1 kb, −3.5 kb, or −5.2 kb upstream of the TSS harbors the potential to drive GFP expression in mosaic animals in several tissues including liver at 6 dpf (Figure S3C, S3E). Robust expression in the liver was confirmed in animals harboring stable germ-line incorporation of these transgenes (Figure S3D, S3F). However, these *angptl4* upstream regulatory sequences were not sufficient to drive detectable reporter expression in the intestine (Figure S3G) or islet (data not shown). We therefore reasoned that information governing transcription in the intestine and islet must be located distal to the TSS and proximal promoter.

### Multiple *angptl4* intronic regulatory modules confer tissue-specific transcription

**Figure 3:**
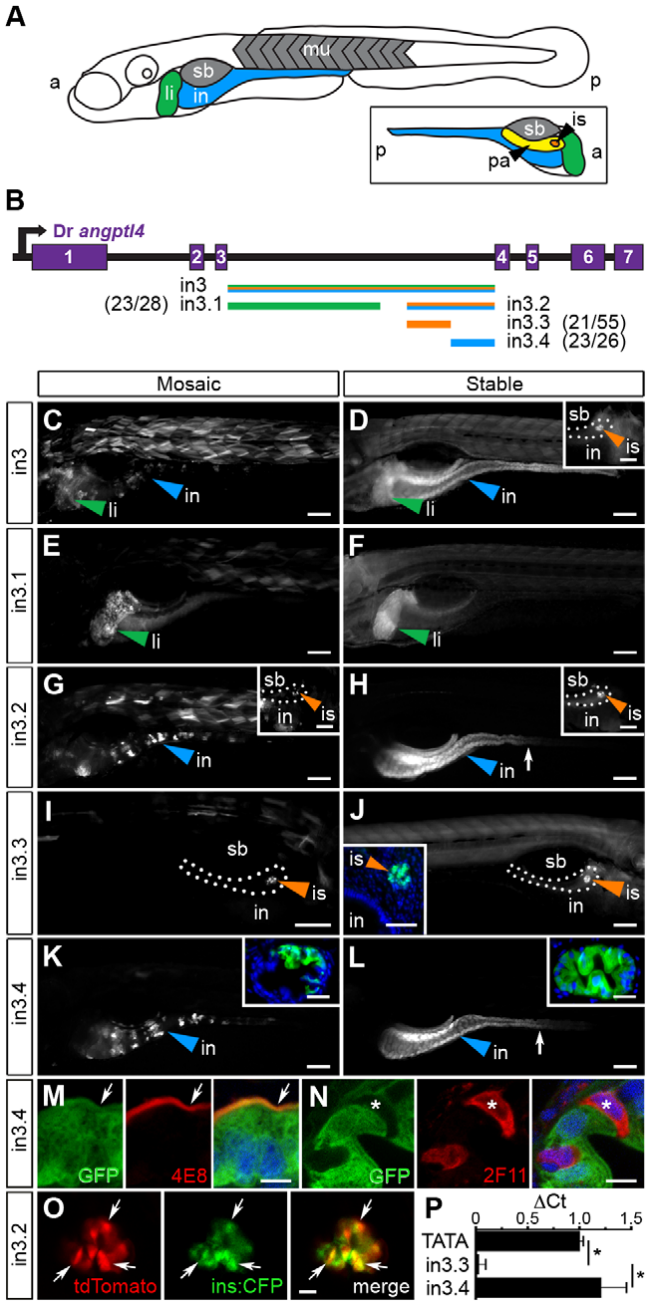
Non-overlapping regulatory modules within *angptl4* intron 3 confer liver, islet, and enterocyte-specific reporter expression. (A) Depiction of the 6 dpf zebrafish showing liver (li, green), intestine (in, blue), swim bladder (sb, grey), and muscle (mu, grey), with the fish oriented anterior (a) to the left and posterior (p) to the right. The opposite orientation reveals the exocrine pancreas (pa, yellow) and islet (is, orange). (B) Scaled schematic of the zebrafish *angptl4* locus and non-coding DNA assayed for regulatory potential. Modules are color coded according to the tissues in which they confer expression. Ratios of islet or intestine positive fish versus total fish expressing gfp are shown in parentheses next to truncation labels. (C–N) Representative images of GFP reporter expression in mosaic (column 1) and F_1_ stable (column 2) animals driven by each non-coding DNA region (rows). Scale bars = 100 µm; li = liver, is = islet, in = intestine, sb = swim bladder. Colored arrowheads indicate tissue with specific reporter expression. (C–D) Full-length intron 3 (in3; 2,136 bp) is sufficient to promote expression of the reporter in the liver, islet (D, inset, scale bar = 50 µm), and intestine. (E–F) Truncation in3.1 (1,219 bp) confers expression in the liver. (G–H) Truncation in3.2 (701 bp) confers expression in both the intestine and islet (H, inset). Inset scale bar = 50 µm. (I–J) Truncation in3.3 (387 bp) confers islet expression. A transverse section (inset, J) reveals islet expression (nuclei stained with DAPI). Inset scale bar = 50 µm. (K–L) Truncation in3.4 (316 bp) confers intestinal expression. Insets in panels K and L contain transverse sections showing expression localized to the intestinal epithelium (nuclei stained with DAPI). Inset scale bar = 25 µm. The dotted lines in panels D, G, H, and I outline the pancreas. The white arrows in panels H, K, and L mark the boundary between the anterior intestine (segment 1) and mid-intestine (segment 2). (M–N) Cells expressing GFP driven by the in3.4 regulatory module colocalize with a marker (4E8, red, white arrow) of the brush border of absorptive enterocytes, but fail to co-localize with marker for secretory cells (2F11, red, asterisk). Nuclei stained with DAPI. Scale bars = 5 µm. (O) Intercross of *Tg(in3.2-Mmu.Fos:tdTomato)* with β-cell specific reporter line (*Tg(ins:CFP-NTR)^s892^*) show colocalization of tdTomato and CFP in the islet. Scale bars = 10 µm. (P) Quantitative PCR shows that the in3.4 module and the *angptl4* promoter (TATA box), but not the in3.3 module, are hypersensitive to DNase I cleavage in intestinal epithelial cells isolated from adult zebrafish. Asterisks denote P-value<.01 from unpaired T-tests between TATA box or in3.4 and in3.3 regions. Error bars represent standard deviation from four biological replicates using cells pooled from 3 wild-type adult zebrafish per replicate.

Relatively high levels of DNA sequence conservation in both teleost and mammalian lineages (Figure 2) prompted us to test the 3^rd^ intron of zebrafish *angptl4* for transcriptional regulatory potential. We cloned full-length zebrafish *angptl4* intron 3 (2,136 bp; designated in3) into a Tol2 transposon reporter vector upstream of a minimal mouse *Fos* promoter (*Mmu.Fos*) driving transcription of a GFP or tdTomato reporter. Importantly, the minimal *Fos* promoter alone is relatively inactive in most tissues and is not sufficient to drive transcription of detectable levels of GFP in the intestine, islet, or liver [50]. Analysis of 6 dpf zebrafish larvae with mosaic expression of the *Tg(in3-Mmu.Fos:GFP)* transgene disclosed that full-length in3 is sufficient to confer reporter expression in multiple tissues including the liver, muscle, intestine (Figure 3C), and islet (not shown). This expression pattern was confirmed in fish with stable germ-line incorporation of the transgene (Figure 3D). Guided by sequence conservation between zebrafish and medaka (Figure 2A), we assayed serial truncations of in3 for spatial regulatory potential to determine whether reporter transcriptional activity in these distinct tissues is governed by the same CRM or through multiple discrete CRMs, (Figure 3B). The first truncation separated liver expression (1,219 bp, designated in3.1, Figure 3E, 3F) from islet and intestinal expression (701 bp; designated in3.2, Figure 3G, 3H). Further truncation of in3.2 uncoupled islet (387 bp; designated in3.3; Figure 3I, 3J) and intestinal (316 bp; designated in3.4; Figure 3K, 3L) expression. This analysis therefore revealed non-overlapping modules sufficient to confer mosaic and stable reporter expression in the liver, islet, and intestinal epithelium that is consistent with endogenous *angptl4* mRNA expression (Figure 1).

We next sought to identify the specific cell types in the intestinal epithelium and pancreatic islet in which modules in3.3 and in3.4 respectively enhance transcription. To define the cell type within the islet in which module in3.3 is active, we utilized a zebrafish transgenic line that drives expression of cyan fluorescent reporter (CFP) specifically in insulin-producing β-cells within the islet (*Tg(ins:CFP-NTR)^s892^*) [60]. *In vivo* imaging of 6 dpf progeny from intercrosses of *Tg(ins:CFP-NTR)^s892^* and *Tg(in3.2-Mmu.Fos:tdTomato)* adults revealed strong co-localization of CFP and tdTomato (Figure 3O), indicating that the in3.3 module specifically enhances transcription in pancreatic β-cells. Immunofluorescence assays of sectioned 6 dpf zebrafish stably expressing the *Tg(in3.4-Mmu.Fos:GFP)* transgene revealed that GFP driven by the in3.4 module co-localizes with 4E8-positive absorptive enterocytes (Figure 3M) but not with 2F11-positive secretory cells in the intestinal epithelium (Figure 3N). These data suggest that in3.4 functions as an enterocyte-specific transcriptional regulatory module.

We next tested whether the intestine-specific reporter expression generated by module in3.4 is independent of the *Fos* minimal promoter, orientation, and proximal position to the TSS. This module is located downstream of the TSS in intron 3 of the endogenous *angptl4* gene; however, our synthetic reporter construct positions it upstream of the TSS and the *Fos* minimal promoter. We therefore cloned in3.4 into a position downstream of *GFP* in either the forward or inverse orientation under control of either a *Fos* minimal promoter or the −1 kb *angptl4* promoter. Each of these constructs was sufficient to promote robust reporter expression in the anterior intestine of 6 dpf mosaic and stable zebrafish (Figure S4A and data not shown), similar to our observations with in3.4 located in the proximal position (Figure 3K, 3L). These results establish that in3.4 is a bona fide transcriptional enhancer module active in enterocytes in the anterior intestine.

We next used DNase I hypersensitivity to determine if the in3.4 module functions as an intestinal regulatory module *in vivo* at the endogenous *angptl4* locus. To obtain a sufficient number of intestinal epithelial cells for this assay, we analyzed intestines from adult zebrafish. Stable transgenic zebrafish harboring the in3.2 or in3.4 reporter maintain reporter activity in the intestine into adulthood (Figure S4B and data not shown) indicating this module and associated *trans*-regulators are active in the adult zebrafish intestine. We find that the endogenous *angptl4* promoter and in3.4 module, but not the adjacent in3.3 module, are hypersensitive to DNase I cleavage in intestinal epithelial cells isolated from adult zebrafish (Figure 3P). The endogenous in3.4 module is therefore an active regulatory module in the intestinal epithelium, under regulatory control distinct from the adjacent in3.3 module, consistent with our transgenic reporter analysis of this same region. Together, these data reveal extensive transcriptional regulatory potential within intron 3 of zebrafish *angptl4* and suggest that distinct intronic modules may mediate spatially restricted transcription of *angptl4* in the intestinal epithelium, pancreatic β-cells, and liver.

### Evolution of the islet and intestinal regulatory modules

**Figure 4:**
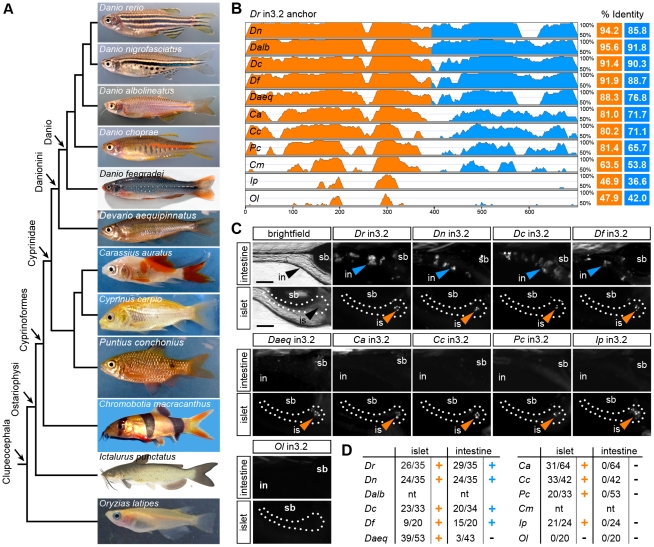
Functional evolution of the islet and intestinal regulatory modules in 12 fish species. (A) Unscaled phylogram based on information from [58], [59] showing images and relative relationships of 12 fish for which intronic sequences were analyzed. *Danio rerio* (*Dr*, zebrafish), *Danio nigrofasciatus* (*Dn*), *Danio albolineatus* (*Dalb*), *Danio choprae* (*Dc*), *Danio feegradei* (*Df*), *Devario aequipinnatus* (*Daeq*, giant danio), *Carassius auratus* (*Ca*, goldfish), *Cyprinus carpio* (*Cc*, carp), *Puntius conchonius* (*Pc*, rosy barb), *Chromobotia macracanthus* (*Cm*, clown loach), *Ictalurus punctatus* (*Ip*, channel catfish), *Oryzias latipes* (*Ol*, medaka). (B) VISTA plot displaying the global pairwise alignment of orthologous in3.2 regions from each species anchored to zebrafish (*Dr*) in3.2. Orange peaks correspond to regions in the alignment that correspond to *Dr* in3.3 (islet module). Blue peaks correspond to regions in the alignment that correspond to *Dr* in3.4 (intestine module). Percent identity is calculated from pairwise alignments of each module with zebrafish (VISTA parameters: 25 bp sliding window, LAGAN alignment). (C) Representative islet and intestinal images from injections of each orthologous in3.2 module. Orange or blue arrowheads mark positive islet or intestine expression, respectively. The absence of arrowheads denotes negative expression in each tissue. (D) Summary of mosaic expression for each species. Ratios of islet or intestine positive fish versus total fish expressing gfp are shown. Orange or blue (+) denotes that the construct was sufficient to confer expression in the islet or intestine, respectively. Black (−) denotes insufficiency. Note that *Dalb* and *Cm* sequences were not tested (nt) in this heterologous functional assay. See also Figures S5 and S6.

We used comparative genome sequence analysis from 12 teleost fishes and heterologous *in vivo* reporter assays to explore the evolution of the islet and intestinal regulatory modules. We originally postulated that evolutionary conservation of non-coding sequences could be used to predict the location of *cis*-regulatory regions controlling spatial and environmental regulation of *angptl4* transcription (Figure 2). However, the significant amount of time (approximately 230–307 million years ago) [59] since the divergence between zebrafish (clade Otocephala; order Cypriniformes) and the other teleost fish with available genome sequence (all from clade Clupeocephala, such as medaka) did not permit high-resolution analysis of recent evolution of zebrafish *angptl4* regulatory sequences (Figure 2A). We therefore sequenced the intronic region orthologous to in3.2 from 10 additional Ostariophysi species, including 1 from order Siluriformes (channel catfish, *Ictalurus punctatus*) and 9 other members of order Cypriniformes (Figure 4A). Because genome sequences are not currently available for these species, we took advantage of the intronic location of these regulatory modules by utilizing PCR primers targeting highly conserved sequences in flanking exons 3/4 or intron 3 to clone and sequence these putative regulatory regions. As expected, pairwise alignments of new sequences orthologous to zebrafish in3.2 revealed an inverse relationship between the phylogenetic distance between the two species and module sequence conservation, with the intestinal module diverging more rapidly than the islet module (Figure 4B, Figures S5 and S6). To test the functional consequences of the observed module divergence in these teleost species, we analyzed each module using our zebrafish mosaic transgenic assay for regulatory potential in the intestine and islet. Despite accounts of functional conservation in the absence of primary sequence conservation [50], [61], the non-coding sequence within medaka *angptl4* intron 3 orthologous to zebrafish in3.2 (Ol in3.2) failed to drive reporter expression in either the intestine or islet (Figure 4C). Notably, all tested Ostariophysi modules elicited robust reporter expression in the islet (Figure 4C). However, only in3.2 from Cypriniformes species within the Danio monophyletic group (*Danio nigrofasciatus, D. choprae, D. feegradei*) [62], [63] were sufficient to confer reporter expression in the intestine (Figure 4C) despite marked regions of sequence conservation within the intestinal module in other Cypriniformes species (*D. aequipinnatus*, *C. auratus*, *C. carpio*, *P. conchonius*). These results reveal differential evolutionary dynamics of the *angptl4* intestinal and islet modules and support the hypothesis that high sequence conservation is required for tissue-specific transcription.

### Truncation mapping of the islet and intestinal regulatory modules in *angptl4* intron 3

**Figure 5:**
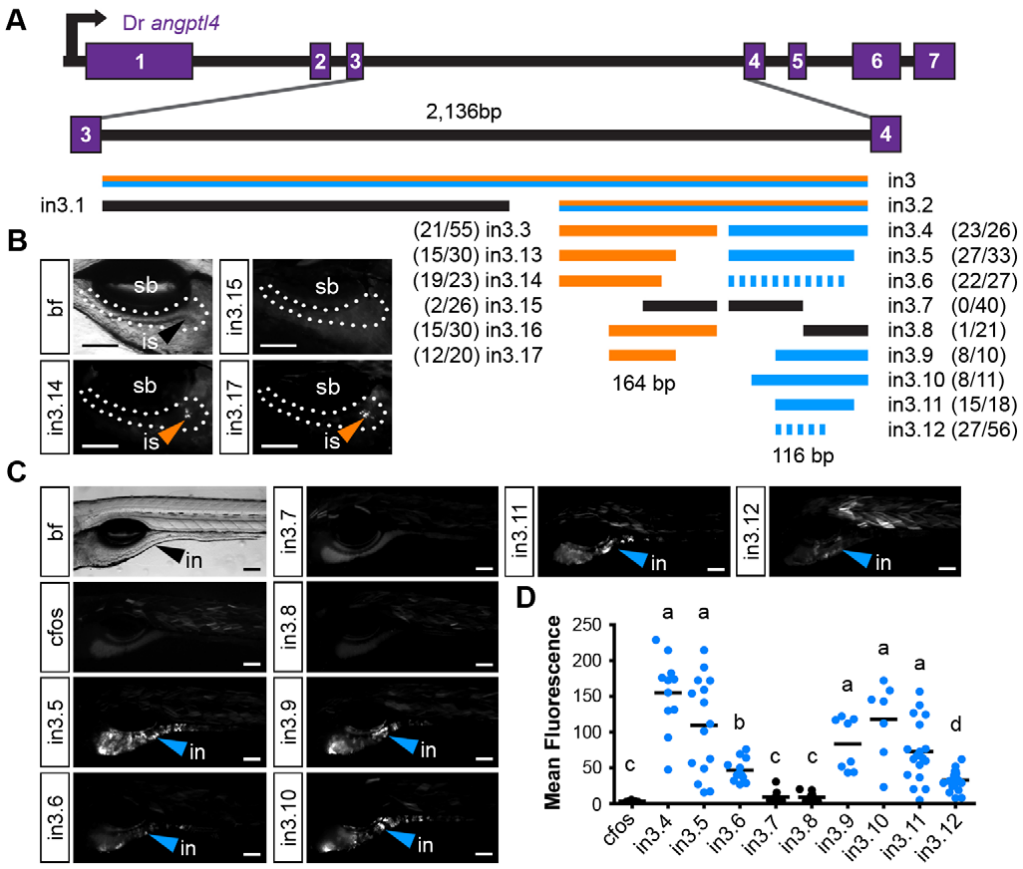
Truncation mapping of the islet and intestinal regulatory module. (A) Scaled schematic of the zebrafish *angptl4* locus showing annotations of truncations assayed for regulatory potential. Orange lines indicate sufficiency to confer islet expression, blue lines indicate sufficiency to confer intestinal expression, and black lines indicate insufficiency in intestine and islet. Dashed blue lines indicate reduced intestinal expression compared to in3.4. Ratios of islet or intestine positive fish versus total fish expressing gfp are shown in parentheses next to truncation labels. (B) Representative images of islet views from mosaic injected fish of each truncation construct. Orange arrows mark islet expression (is). Scale bars = 100 µm. (C) Representative images of intestinal views from mosaic fish injected with each truncation construct. Blue arrows mark intestinal expression (in). Scale bars = 100 µm. (D) Relative mean intestinal fluorescence within the intestine was quantified in mosaic animals (see Materials and Methods) and plotted per injected fish. Circles represent mean fluorescence averaged for three mosaic patches within one fish, and are colored blue or black to designate truncations that are sufficient or insufficient to confer intestinal expression, respectively. Statistical significance was tested using Kruskal-Wallis one-way analysis of variance (labels: a = P<.001, b = P<.05 vs. *Fos*; c = P<.001, d = P<.01 vs. in3.4). Scale bars = 100 µm.

Guided by our conservation analyses, we next sought to map the boundaries of critical regulatory regions in the zebrafish in3.3 islet and in3.4 intestinal CRMs by creating and testing truncations of these modules. Each truncation construct was injected into embryos and analyzed at 6–7 dpf for mosaic expression in the islet or intestine. These analyses defined a 164 bp region sufficient to confer islet expression (in3.17; Figure 5A, 5B) including a 129 bp region present in all islet-sufficient truncations (Figure 5A). This 129 bp region overlaps with conserved regions identified in our comparative evolutionary analysis (Figure 7A). *In silico* prediction of transcription factor binding sites in this critical region identified putative binding sites for multiple transcription factors known to be active in pancreatic islets such as Myc [64], [65] and Arnt/HIF1b [66], [67], as well ubiquitously expressed transcription factors with important regulatory roles in β-cells such as USF [68] and CREB/ATF [69] (Figure 7A).

A distinct 116 bp region (in3.12) was found to be sufficient to confer intestinal expression (Figure 5A, 5C). Notably, the intensity driven by in3.12 in the intestine was lower than other larger truncations of this module that confer strong intestine-specific expression, such as in3.9 and in3.11 (Figure 5C, 5D). The in3.12 truncation therefore represents a minimal intestinal regulatory module that requires additional flanking sequence information to facilitate maximal activity. Intriguingly, the in3.11 truncation, which displays strong intestinal activity, overlaps with two regions of high conservation identified in our comparative evolutionary analysis (Figure 7B), suggesting that specific sequences within these conserved regions may be responsible for mediating intestine-specific enhancer activity. Together, these results define the approximate boundaries of functional regulatory DNA within *angptl4* intron 3 required for intestinal and islet transcription.

### Site-directed mutagenesis confirms functional motifs within the intestinal module

**Figure 6:**
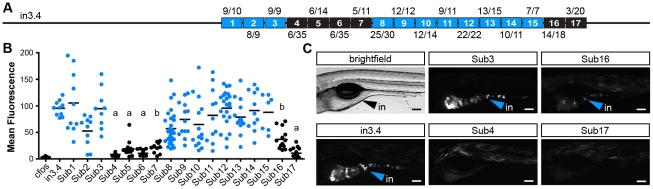
Site-directed mutagenesis defines DNA motifs required for intestinal expression. (A) Scaled schematic showing 10 bp substitution blocks tiled across the zebrafish *angptl4* in3.11 region within the context of the entire in3.4 intestinal module. Black or blue blocks represent mutations that do or do not significantly alter intestinal expression compared to wild type in3.4, respectively (see below). Ratios of intestine positive fish versus total fish expressing GFP are shown in parentheses above or below substitution block labels. (B) Relative mean intestinal fluorescence was quantified in mosaic animals (see Materials and Methods) and plotted per injected fish. Circles represent mean fluorescence averaged for three mosaic patches within a single fish and are colored blue or black to designate mutations that do or do not confer intestinal expression, respectively. Statistical significance was tested using Kruskal-Wallis one-way analysis of variance (labels: a = P<.01 vs. in3.4, P>.05 vs. *Fos*; b = P>.05 vs. *Fos*; unlabeled = P>.05 vs. in3.4, P<.01 vs. *Fos*). (C) Images from animals with mosaic expression of five representative mutant constructs are shown. Blue arrows indicate intestinal expression (in). Scale bars = 100 µm.

**Figure 7:**
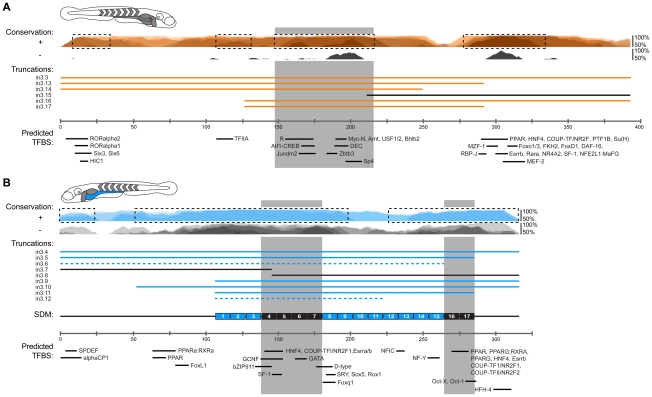
Summary of functional conservation and mapping of islet and intestinal regulatory information. (A) Conservation plots, module truncations, and predicted transcription factor binding sites (TFBS) in islet CRM in3.3 are overlayed and annotated to scale. The grey shaded box represents the region that is present in all positive truncations and has strong conservation in islet-positive species. (B) Conservation plots, module truncations, SDM data, and predicted transcription factor binding sites (TFBS) in intestinal CRM in3.4 are overlayed and annotated to scale. Two grey shaded boxes represent regions that are present in all positive truncations, are required for intestinal expression, and have strong conservation in intestine-positive species. Dotted boxes in panels A and B represent highly conserved regions from each (A) islet-positive or (B) intestine-positive species used to predict common TFBS (see Figures S5 and S6, and Materials and Methods).

To complement our comparative genomic and truncation strategies, we used site-directed mutagenesis (SDM) to generate a higher-resolution understanding of the functional DNA motifs required for enterocyte-specific transcription of *angptl4*. Ten base-pair substitutions were tiled across the region corresponding to in3.11 within the context of the entire in3.4 module, and assayed for competency to drive intestinal transcription (Figure 6A). This analysis revealed two regions of 40 bp and 20 bp that disrupt intestinal reporter expression when mutated (Figure 6B, 6C). DNA adjacent to these regions was not required for intestinal expression, validating the efficacy of the experimental approach. These data support our truncation mapping experiments (Figure 5) by localizing a required region within the in3.12 truncation, as well as a second region within the larger, more active in3.11 truncation. We observed strong overlap between conserved sequences in intestine-positive in3.4 modules identified in our comparative genomic analysis and regions identified by SDM as required for intestinal expression (Figure 7B). Specifically, SDM revealed that regions deleted in *Daeq* and *Dn* lineages do not harbor functional motifs required for intestinal expression. Most notably, mutation block 4–7 overlap with the single nucleotide polymorphisms between Devario and Danio species (Figure S6). This region harbors predicted binding sites for transcription factors involved in intestinal epithelial cell biology (Figure 7B; see Discussion) that represent attractive candidates for controlling enterocyte-specific *angptl4* transcription.

### The in3.4 module recapitulates *angptl4* suppression by the microbiota

**Figure 8:**
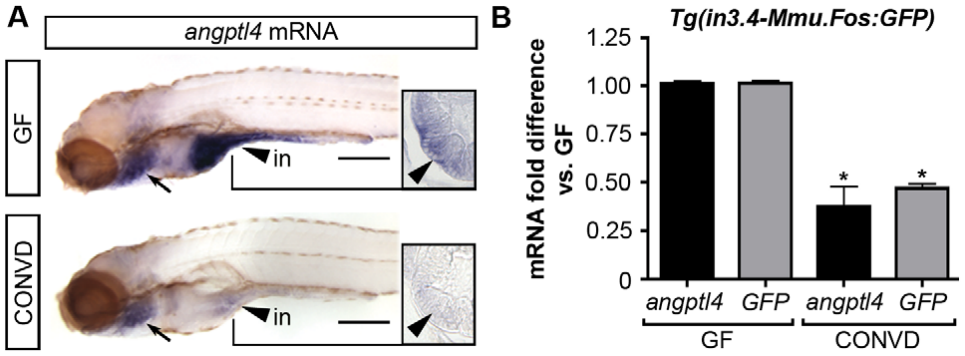
The intestinal module in3.4 recapitulates microbial suppression of *angptl4*. (A) Semi-quantitative whole mount *in situ* hybridization of *angptl4* mRNA in 6 dpf germ-free (GF) and conventionalized (CONVD) animals. Arrowheads mark intestinal expression. Note that the background staining in the gills (arrows) is similar in GF and CONVD fish. Transverse sections show that microbial suppression of *angptl4* mRNA is specific to the intestinal epithelium. (B) Quantitative RT-PCR of *angptl4* and *GFP* mRNA levels in 6 dpf GF and CONVD *Tg(in3.4-Mmu.Fos:GFP)* animals. GF and CONVD animals were derived from the same *Tg(in3.4-Mmu.Fos:GFP)* stable line. *GFP* and *angptl4* mRNA were normalized to *18S* rRNA levels and are shown as fold difference compared to GF controls averaged across 3 experimental replicates ± SEM (2 biological replicate groups of 10 larvae per condition per experiment). Similar results were attained when normalized to *ribosomal protein L32* (*rpl32*) rRNA levels. Asterisks denote P-value<.01 from unpaired T-test between GF and CONVD conditions for each gene. See also Figure S8.

The presence of commensal gut microbiota in mice results in decreased levels of *Angptl4* transcript specifically in the intestinal epithelium, which is thought to lead to increased peripheral fat storage [8]. However, it remained unknown whether this microbe-induced change in transcript levels was due to alterations in transcriptional activity or transcript stability. We speculated that the intestine-specific *cis*-regulatory module within intron 3 could impart this environmental response in the zebrafish. Our previous comparisons of 6 dpf GF zebrafish to age-matched ex-GF zebrafish colonized since 3 dpf with a normal microbiota (conventionalized or CONVD) indicated that the presence of a microbiota results in reduced *angptl4* transcript levels [47]–[49]. To define the cellular origins of this response in zebrafish hosts, we used semi-quantitative WISH assays to reveal marked reduction of *angptl4* mRNA in the intestinal epithelium in 6 dpf CONVD zebrafish compared to age-matched GF controls (Figure 8A). These results indicate that intestinal epithelial suppression of *angptl4* expression is a conserved response to the microbiota in zebrafish and mammalian hosts.

We next tested the ability of the zebrafish intestinal CRM in3.4 to mediate the observed microbial suppression of the endogenous *angptl4* gene. We reared stable *Tg(in3.4-Mmu.Fos:GFP)* zebrafish to 6 dpf under GF or CONVD conditions and assayed transcript levels for both *GFP* reporter and endogenous *angptl4* using qRT-PCR. Consistent with our WISH results, endogenous *angptl4* transcript levels were significantly and reproducibly reduced in CONVD compared to GF animals (Figure 8B). Strikingly, transcript levels of the *GFP* reporter gene were similarly reduced in CONVD compared to GF animals (Figure 8B). These observations were confirmed using an independent stable transgenic line, *Tg(in3.2-Mmu.Fos:tdT)*, harboring the in3.2 reporter which includes the in3.4 module (Figure S7). These data identify the *angptl4* in3.4 module as a nodal *cis*-regulatory module that integrates transcriptional regulatory input from intestinal epithelial-specific and microbial factors.

## Discussion

### Non-overlapping modules confer cell-type specific transcription of *angptl4*

Transcriptional regulation is a key determinant of gene function in the context of animal development and physiology. Recent biochemical and genetic studies in mouse and humans have identified Angptl4 as a critical hormonal regulator of TG-rich lipoprotein metabolism, angiogenesis, and tumor cell survival and metastasis. An improved understanding of the mechanisms controlling Angptl4 activity levels could therefore lead to new approaches for controlling multiple pathophysiologic processes. Although we have a working understanding of Angptl4's post-translational functions, our current knowledge of the mechanisms underlying *Angptl4* transcription in different tissues is relatively limited. Here, we exploited the advantages of the zebrafish model system to examine the regulatory potential of DNA at the *angptl4* locus in all cell types simultaneously and within an intact and living vertebrate organism that can be raised under gnotobiotic conditions. We found that the zebrafish *angptl4* ortholog is expressed in many of the same tissues and cell types as mammalian *Angptl4* (i.e., liver, pancreatic β-cells, and intestinal enterocytes). This finding suggests that the tissue-specific pattern of *Angptl4* expression may have been conserved in the last common ancestor of mammalian and teleost lineages and might have important functional consequences on vertebrate physiology.

Our results reveal that transcription of *angptl4* in distinct tissues might be governed by independent *cis*-regulatory mechanisms. This modular design could have important implications for Angptl4 evolution and function. First, tissue-specific CRMs could have allowed independent evolution of CRM sequence structure. Consistent with this notion, we observed evidence of differential evolution of the islet and intestinal modules within teleost fish lineages (Figure 4). Differential selective pressures influencing CRM sequence evolution likely arise from the vastly different cellular contexts and exogenous stimuli of each cell type. Pancreatic β-cells are surrounded by other endocrine and exocrine pancreatic cells as well as vascular endothelial cells, whereas intestinal epithelial cells are exposed to complex and variable contents of the intestinal lumen and to the cells of the underlying lamina propria. Combining the observations that (i) functional conservation of the intestinal module is restricted to Danio species, (ii) transcriptional activity of the intestinal module is sensitive to the microbial status of the intestinal lumen, and (iii) this microbial regulation of *angptl4* transcript levels is conserved in mammals, suggests an intriguing possibility that genes expressed in intestinal epithelia exposed to the dynamic and potentially hazardous luminal environment undergo relatively rapid regulatory evolution. Previous studies have suggested that the expression and function of *defensin* genes within the epithelia of the intestine and other exposed tissues has driven rapid evolution of their coding sequences [70], and our results raise the possibility that similar selective pressures may also affect evolutionary rate of regulatory sequences for *angptl4* and potentially other genes. Second, discrete *cis*-regulatory modules could have led to the independent evolution of Angptl4 synthesis in each respective cell type. This evolution would allow each expressing cell type to independently communicate its physiologic status and environmental exposures systemically by secreting Angptl4 into circulation, and locally by secreting Angptl4 into the extracellular space. The modular organization of these independent tissue-specific CRMs suggests that therapeutic strategies could be developed to control Angptl4 synthesis in specific target tissues without unintended effects on Angptl4 synthesis in other tissues.

Previous studies of CRM evolution in vertebrates and invertebrates have focused primarily on enhancers regulating expression of genes involved in development [50], [61], [71]. These studies revealed that maintenance of regulatory function can be sustained over long evolutionary distances despite marked sequence dissimilarity and turnover of regulatory information. Our work provides a novel example of utilizing genomic DNA sequences from both close and distant relatives to define the evolutionary dynamics of multiple CRMs and marks the first time to our knowledge that such an extensive exploration (i.e., 12 related fish species) was carried out in a vertebrate. We find that transcriptional output generated by both the intestinal and islet modules is maintained through a striking conservation in DNA sequence throughout the entire functional module, with little or no turnover of predicted binding sites. This finding suggests that these modules can comply with the “enhanceosome model” of regulatory information organization, as opposed to the “billboard model,” which accommodates variation in binding site order, orientation, and spacing [72], [73]. However, we detected little non-coding sequence conservation between zebrafish *angptl4* and mouse *Angptl4* intron 3, and we did not detect islet or intestinal reporter expression in a heterologous assay in which we tested full and truncated versions of mouse introns 3 and 4 in the zebrafish (data not shown). This result suggests either that regulatory information governing islet and intestinal expression of murine *Angptl4* is not located within intron 3 or that compensatory *cis*/*trans* mutations render murine intron 3 sequences non-functional in the zebrafish. We suspect that rules governing CRM function and evolution are dependent on the distinct nature of the organism, the specific module, and the signals that the module integrates. It therefore remains an intriguing question as to what extent lessons learned from developmental gene regulation are applicable to the evolution of CRMs controlling expression of genes like *Angptl4* that function in homeostatic physiology or in response to environmental factors like the microbiota [72].

Analyses of Drosophila genomes have elegantly shown that CRM “discovery power scales with the divergence time and number of species compared” [74], and our results suggest that the same will be true in vertebrate lineages. Moreover, our data underscore the need for more reference genome sequences from phylogenetically diverse fish species, in combination with experimentally tractable fish models such as the zebrafish, to facilitate new insights into vertebrate CRM function and evolution.

### The nature of microbial signals regulating intestinal transcription of *angptl4*

The intestinal microbiota has been identified as an important environmental factor that contributes to host energy storage and obesity, and our results provide critical new insights into how this might be achieved. Previous studies in gnotobiotic mice have shown that the intestinal microbiota regulates fat storage in part by suppressing *Angptl4* transcript levels in the epithelium of the small intestine but not in liver or WAT [8], [11]. However, it remained unclear whether these microbe-induced reductions of *Angptl4* transcript levels were due to alterations in *Angptl4* transcription or mRNA turnover. Furthermore, the molecular basis of the intestinal specificity of this response remained unknown. Our results reveal that zebrafish *angptl4* transcript levels are also reduced in the intestinal epithelium in the presence of a microbiota, suggesting that the microbial regulation of *angptl4* transcript levels might be an evolutionarily ancient feature of host-microbe commensalism in the vertebrate intestine. Our observation that transcript levels from the in3.4 reporter and the endogenous *angptl4* gene respond similarly to microbial colonization strongly suggests that the microbiota regulates *angptl4* expression, at least in part, by reducing the transcriptional activity of this enterocyte-specific enhancer module. These results indicate that enterocyte-specific and microbial control of *angptl4* transcription is conferred through a shared intronic enhancer.

Future investigation will be required to determine whether microbial regulation of in3.4 activity is achieved by (i) reducing the accessibility of this chromatin region to activating *trans*-factors, (ii) subverting the expression or activity of activating *trans*-factors, and/or (iii) inducing expression or activity of repressive *trans*-factors that function through this module. To distinguish between these models, it will be useful to identify the microbial activity and host transcription factors that regulate *angptl4* transcription in the intestinal epithelium. We previously reported that colonization of GF zebrafish with a microbiota harvested from conventionally raised zebrafish or mice resulted in similar suppression of *angptl4* transcript levels in the digestive tract [48]. This finding suggests that the microbial factor(s) regulating zebrafish *angptl4* transcription is expressed by the ‘native’ zebrafish microbiota and in the ‘non-native’ and compositionally distinct mouse gut microbiota. Previous studies have identified individual microbial species sufficient to regulate *angptl4* expression in gnotobiotic zebrafish [47], [48] and mouse hosts [9], [75] as well as in cultured colon cancer cells [31], [76], suggesting that reductionist approaches in these microbial species could be used to define the specific factors they utilize to control expression of *angptl4* homologs and other host genes.

### Potential transcription factors regulating intestinal expression of *angptl4*

In this study, we define two minimal regions within the in3.4 CRM that harbor regulatory activity in the intestine and are also conserved within the Danio lineage (Figure 7B). Predicted transcription factor binding sites within these regions intimates potential roles for these factors in regulation of *angptl4* tissue-specific transcription and/or microbial suppression. Because sequence-specific transcription factors typically recognize 6–12 bp motifs [77], it is reasonable to assume that multiple factors cooperate to combinatorially regulate intestinal expression through this CRM. The Hnf4 family of fatty acid-regulated nuclear receptors has evolutionarily conserved roles in lipid metabolism [78], [79], and Hnf4a is expressed in the intestinal epithelium of zebrafish [80] and mouse [81]. Similarly, GATA factors 4, 5, and 6 are all expressed in the zebrafish [82], [83] and mouse [84], [85] intestinal epithelium and have proposed roles in regulating epithelial cell differentiation. Notably, *C. elegans* GATA family member *elt-2* has been implicated in mediating intestinal epithelial cell immune responses [86], suggesting that GATA factors could mediate tissue-specific as well as microbial regulatory inputs at *angptl4*. PPAR family members have been identified as key regulators of mammalian *Angptl4* expression in adipocytes and hepatocytes through PPAR responsive elements located in the 5′ portion of human *ANGPTL4* intron 3 [27], [30], and zebrafish PPARγ [87] and PPARδ [88] homologs are expressed in the larval intestine. The zebrafish *angptl4* locus contains multiple predicted PPRE sites, including several in both the 5′ and 3′ portion of intron 3 [89]. Most notably, a predicted PPRE was detected within the substitution blocks 16/17 in the intestinal enhancer in3.4 (Figure 7B). However, the PPREs within zebrafish *angptl4* intron 3 that display the highest sequence homology to the defined human *ANGPTL4* intron 3 PPRE mapped outside of minimal regions for either intestinal or islet expression within the 5′ liver module (data not shown). The location of these PPREs in the 5′ region of zebrafish *angptl4* intron 3, combined with the fact that the PPREs discovered in human *ANGPTL4* are also located in the 5′ portion of intron 3, suggests that the predicted PPREs within the 3′ islet and intestine CRMs of zebrafish *angptl4* could represent novel elements for which functional equivalents have not been identified in mammals.

Although these predicted factors represent candidates for controlling intestine-specific regulation of *angptl4*, databases of predicted TFBSs are incomplete and commonly produce both false-positive and false-negative predictions. Moreover, critical regions identified by SDM might reflect sequences that alter nucleosome positioning or histone modification patterns rather than binding sites for sequence-specific transcription factors. Therefore, we anticipate that unbiased methods for transcription factor discovery will provide the most rigorous approach to an improved understanding of this *cis*/*trans* system. The structure-function analysis of the zebrafish in3.4 intestinal enhancer module reported here was designed to identify sequences critical for intestinal activity. It will therefore be interesting to determine whether exogenous microbial inputs are interpreted through the same or distinct motifs within this CRM and how the endogenous *trans*-acting factors mediating microbial and intestinal regulatory inputs interact to determine transcriptional output.

## Materials and Methods

### Zebrafish husbandry

All experiments using zebrafish were performed in wild-type TL or *Tg(ins:CFP-NTR)^s892^* [60] strains according to established protocols approved by the Animal Studies Committee at the University of North Carolina at Chapel Hill. New stable transgenic lines genereated in this study are listed in Table S3. Conventionally raised zebrafish were reared and maintained as described [87]. Production, colonization, maintenance, and sterility testing of germ-free zebrafish were performed as described [45], [49].

### Protein sequence analysis

Protein sequences from top BlastP hits to human (*Homo sapiens*, Hs) ANGPTL4 and zebrafish Angptl4 (*Danio rerio*, Dr) were acquired through NCBI or Ensembl and aligned using MUSCLE with default settings [90]. Amino acids highlighted in black represent identical residues in at least 50% of species, whereas amino acids highlighted in grey represent biochemically similar residues (Boxshade). The cleavage recognition sequence and LPL inhibition domain were annotated using information from previous publications [15], [91]. The boundaries of the fibrinogen domain were annotated using *in silico* predictions [92], [93]. Gaps in the alignment resulting from poorly annotated sequences were manually curated using primary DNA sequence and *in silico* translated using ExPASy [94]. The workflow for inferring phylogenetic relationships was performed at http://mobyle.pasteur.fr/cgi-bin/portal.py. A distance matrix was computed using Phylip 3.67 (Protdist, JTT matrix, default settings), and trees were built using the neighbor-joining method. Bootstrap analysis was performed from 1000 replicates. PHYLIP software and the maximum likelihood probability model [95] using default settings were used to confirm the phylogeny inferred using distance methods. See Table S1 for a complete list of protein sequences used in this study.

### DNA sequence analysis

Genomic DNA sequences encompassing 10 kb upstream, including, and 10 kb downstream of the *Angptl4* locus from *Homo sapiens* (GRCh37:19:8419011:8449257:1), *Mus musculus* (NCBIM37:17:33900702:33928520:−1), *Canis familiaris* (BROADD2:20:55933601:55958821:1), *Danio rerio* (Zv9:2:23312551:23337293), *Oryzias latipes* (MEDAKA1:17:6095931:6120384:1), *Takifugu rubripes* (FUGU4:scaffold_212:367815:391593:1), and *Tetraodon nigroviridis* (TETRAODON8:15:3989265:4012887:1) were acquired through Ensembl. 10 kb was chosen as a cutoff because of proximity to neighboring gene loci. Genomic DNA sequence encompassing the *angptl4* locus from *Danio albolineatus* was generously provided by David Parichy (Department of Biology, University of Washington). For species without available genomic sequence, *angptl4* intron 3 regions were PCR amplified from the relevant genomic DNA using a high-fidelity Taq polymerase (Platinum, Invitrogen) and the primers listed in Table S2. PCR products were cloned into TOPO vector pCR2.1 (Invitrogen) prior to sequencing with M13F primers. An EST corresponding to an *angptl4* homolog in *Ictalurus punctatus* (CK419825) was used to design primers targeting exon 3 and exon 4 for PCR amplification of the full-length intron 3. For Cypriniformes species, ESTs EG548328 (*Rutilus rutilus*), DT085020 (*Pimephales promelas*), GH715226 (*Pimephales promelas*), and AM929131 (*Carassius auratus*) were aligned and used to design primers targeting highly conserved regions in *angptl4* exon 3 and exon 4, which we predicted would function for multiple Cypriniformes species. These primers were used to amplify, clone, and sequence the full-length intron 3 from *Cyprinus carpio* and *Chromobotia macracanthus*. Alignment of *Cc*, *Cm*, and *Dr* revealed 100% conservation at the extreme 5′ end of the in3.2 module. We used a forward primer targeting in3.2 in combination with a reverse primer targeting exon 4 for cloning of the remaining Cyprinidae species. The bacterial artificial chromosome golwb118_K01 containing the *angptl4* locus from *Oryzias latipes* was provided by Hiroyo Kaneko (Laboratory of Bioresource, National Institute for Basic Biology, Okazaki, Japan). *Carassius auratus*, *Puntius conchonius*, *Cyprinus carpio*, *Devario aequipinnatus*, and *Chromobotia macracanthus* genomic DNA was extracted from the fins of two individuals acquired from commercial suppliers. Genomic DNA from *Ictalurus punctatus* and Danio species (*Danio nigrofasciatus*, *Danio choprae*, *Danio feegradei*) from one individual were generously provided by Zhanjiang Liu (Department of Fisheries and Allied Aquacultures, Auburn University) and David Parichy (Department of Biology, University of Washington), respectively. Novel *angptl4* intron 3 sequences generated in this study were deposited in GenBank with accession numbers JN606312–JN606321. Intronic sequences were aligned in mVISTA using LAGAN [96] and visualized using VISTA conservation plots (100 bp windows Figure 2 and 25 bp windows Figure 4) [97].

### Motif and transcription factor binding site (TFBS) predictions

DNA sequences were queried for predicted transcription factor binding sites deposited in TRANSFAC [98] and JASPAR [99] databases using MATCH [100] and TESS [101] programs using default settings. We used a discriminative motif MEME [102] search to discover motifs common to islet-positive or intestine-positive intronic regions, using sequences orthologous to in3.4 or sequences orthologous to in3.3, respectively, as negative selectors. To determine if MEME motifs were unique to islet- or intestine-positive regions, we used MAST [103] to query islet-negative (*Ol* in.3) or intestine-negative (*Daeq, Ca, Cc, Pc, Cm, Ip, Ol* in3.4) sequences for islet-positive or intestine-positive MEME motifs, respectively. TOMTOM [104] was used to query MEME hits against TRANSFAC and JASPAR databases.

### Whole-mount *in situ* hybridization assays

*In situ* hybridization was performed in whole zebrafish as described [87], except that heads and tails were removed from euthanized 17 dpf animals prior to fixation. Sense and anti-sense riboprobes targeting zebrafish *angptl4* were generated by digesting plasmid fj89c07 in pBK-CMV (NCBI Accession XM_686956) with NotI (sense) or BamHI (anti-sense), and transcribed *in vitro* using T3 (sense; Epicentre) or T7 RNA polymerase (anti-sense; Epicentre). Sense riboprobes were used in each experiment as a negative control.

### Quantitative reverse transcription PCR assays

Total RNA was extracted from groups of 6 dpf whole zebrafish larvae from 6 dpf zebrafish (10 larvae per group, 2 biological replicate groups per condition per experiment, 2 experimental replicates total) using TRIzol Reagent (Invitrogen) or the Qiagen RNeasy (Qiagen) kit using manufacturer's protocol. qRT-PCR was performed as described [49]. Primers used in qRT-PCR assays are listed in Table S2.

### Transcription start site and promoter mapping

ESTs at the zebrafish *angptl4* locus were analyzed using UCSC and Ensembl genome browsers. Total RNA was extracted from adult zebrafish intestines and subjected to 5′RACE using the FirstChoice RLM-RACE kit (Ambion), according to the manufacturer's specifications (see Table S2 for primers). Three clones were sequenced and mapped to the zebrafish *angptl4* locus.

### Reporter construct cloning

All PCR reactions used for cloning were performed with high-fidelity DNA polymerase (PfuTurbo, Stratagene; Phusion, Invitrogen; Platinum Taq, Invitrogen) and TOP10 chemically competent *E. coli* (Invitrogen). The bacterial artificial chromosome C177A22 containing the zebrafish *angptl4* locus was used as the template for all zebrafish *angptl4* promoter and intronic PCR amplification and cloning. Mouse BAC (RP24-294G12, CHORI), Medaka BAC (golwb118_K01), and sequenced pCR2.1 clones (*Ip, Pc, Cc, Ca, Daeq, Df, Dc, Dn*) containing intronic regions orthologous to zebrafish in3.2 from each species were used as source material for cloning in heterologous reporter assays. The plasmid pT2cfosGW [50] was used as the vector backbone for all Tol2 transgenic reporter assays. The *Fos* minimal promoter and *angptl4* 5′ upstream regions were PCR amplified and directionally cloned into pT2cfosGW using XhoI and BamHI restriction sites. This step removed both the original *Fos* promoter and the upstream Gateway site. Of note, we observed significant levels of reporter expression in muscle tissue upon removal of the Gateway cloning site (Figure 5C,D and data not shown). Intronic DNA was cloned upstream of the *Fos* minimal promoter in pT2cfosGW using Gateway reagents as described [36]. The intronic module in3.4 was non-directionally cloned into *Tg(-1kbangptl4:GFP)* using the single BglII site located downstream of SV40polyA. A vector (*Tg(in3.4-Mmu.Fos:GFP)*) containing the *angptl4* intronic module in3.4 was used as the source vector for site-directed mutagenesis. To create site-directed substitutions, 50 bp complementary primers containing two 20 bp regions complementary to in3.4, separated by a 10 bp substitution block, were used in circular PCR followed by DpnI treatment to digest methylated parent plasmid. A ClaI restriction site was incorporated into the 10 bp region in order to screen for mutant bacterial colonies. Selection of nucleotide exchange was generally A–C and G–T, except in cases that would create a site amenable to DamI methylation. All plasmids were verified by Sanger dideoxy terminator sequencing. All primers used are listed in Table S2.

### Injections, imaging, and reporter quantification

Co-injections of Tol2 plasmid and transposase mRNA were performed as described [36]. Generally, 100–200 zebrafish embryos were injected at the 1–2 cell stage with approximately 69 pg of plasmid DNA at a DNA∶transposase ratio of 1∶2. Injections of each construct were performed with at least two sequence-verified plasmids in two independent experiments. Mosaic expression patterns were quantified as follows: at least 200 fish were visually observed, and at least 10 were scored per construct for positive/negative expression in selected tissues. At least 7–20 fish/construct were imaged at the same magnification and exposure time and densitometric measures were quantified in 8-bit grey scale images using ImageJ software [105]. Three mosaic patches within a given tissue of an imaged fish were quantified for mean fluorescence intensity and averaged. Statistical significance was analyzed using Kruskal-Wallis one-way analysis of variance and Dunn's multiple comparison test using GraphPad Prism software. Injected larvae were raised to adulthood and screened for stable germ-line insertion. Where indicated, patterns identified in mosaic animals were verified in a least two independent stable germ-line insertions (3). In each case, independent pedigrees of the same Tol2 vector displayed the same specific pattern of expression in the intestine, liver, and islet, respectively.

### Immunohistochemistry

Staining of fixed and sectioned 6 dpf zebrafish was performed exactly as described [49]. Primary antibodies used in this study were anti-GFP (Rabbit, 1∶500, Invitrogen), 2F11 (mouse, 1∶200), 4E8 (mouse, 1∶200; gifts from Julian Lewis), and secondary antibodies were AF568 (goat anti-mouse, 1∶500, Invitrogen) and AF488 (goat anti-mouse, 1∶500, Invitrogen).

### DNase I hypersensitivity

Three intestines were dissected from adult zebrafish at 1 year post-fertilization, splayed, and washed extensively with 1× PBS. Intestines were incubated for 15 minutes on ice in 5 ml of Dissociation Reagent 1 (1× PBS, 30 mM EDTA, 1.5 mM DTT, 1× Complete protease inhibitors; Roche), then transferred to Dissociation Reagent 2 (1× PBS, 30 mM EDTA, 1× Complete protease inhibitors) and shaken at 25°C until epithelial layers were sufficiently sloughed. Epithelial cells were collected, washed in 1× PBS, and re-suspended in 500 microliters of RSB (10 mM Tris-HCl pH 7.4, 10 mM NaCl, 3 mM MgCl_2_). Cells were gently lysed in 10 ml cold RSB plus 0.075% NP-40 and nuclei pelleted at 500× G at 4°C for 10 minutes. Nuclei were incubated with various concentrations of Dnase I (0–1.5 units, NEB) for 10 minutes at 37°C. Reactions were stopped by adding an equal volume of 2× Lysis Buffer (1% SDS, 200 mM NaCl, 10 mM EDTA, 20 mM Tris pH 7.5, 0.4 mg/ml proteinase K) and incubated overnight at 37°C. Digested DNA was extracted using phenol/cholorform/isoamyl alcohol (Fisher), precipitated with ethanol and sodium acetate, and quantified using a fluorimeter (Qubit, Invitrogen). Quantitative PCR was performed as described above using primers listed in Table S2.

## Supporting Information

Figure S1 Phylogeny of Angptl4 and Angptl3 proteins from multiple vertebrate species. Distance phylogram of Angiopoietin-like 3 and 4 from zebrafish (*Dr*, *Danio rerio*), catfish (*Ip*, *Ictalurus punctatus*), medaka (*Ol*, *Oryzias latipes*), tetraodon (*Tn*, *Tetraodoan nigroviridis*), fugu (*Tr*, *Takifugu rubipres*), xenopus (*Xt*, *Xenopus tropicalis*), chicken (*Gg*, *Gallus gallus*), mouse (*Mm*, *Mus musculus*), human (*Hs*, *Homo sapiens*), dog (*Cf*, *Canis familiaris*), pig (*Ss*, *Sus scrofa*), and cow (*Bt*, *Bos taurus*). All nodes are significant (>700/1000 bootstrap replicates) except those marked with an asterisk (*). Phylogenic relationships inferred through Maximum Likelihood yield similar branching with differences only in the positions of the nodes separating Xt Angptl3 and Angptl4 and Gg Angptl3 and Angptl4 from mammals (data not shown). Scale bar indicates phylogenetic distance, in number of amino acid substitutions per site. See Table S1 for protein sequences.
(TIF)

Figure S2 Alignment of Angptl4 proteins from multiple vertebrate species. (A) Multiple sequence alignment of Angptl4 proteins from representative vertebrate species. Amino acids highlighted in black represent identical residues in at least 50% of species, whereas amino acids highlighted in grey represent biochemically similar residues. The green line denotes the cleavage recognition sequence [91], the blue line denotes the experimentally defined LPL inhibition domain [14], and the orange line denotes the *in silico* predicted fibrinogen domain. Black downward arrows designate the exon 2/3 boundary in human, black upward arrows designate the exon2/3 boundary in zebrafish. White downward arrows designate the exon 3/exon 4 boundary in human, white upward arrows designate the exon 3/exon 4 boundary in zebrafish. The black asterisk marks the position of the human E40K variant [16]. (B) Percent identity and percent similarity matrix for each species pair.
(TIF)

Figure S3 Non-coding DNA upstream of the zebrafish *angptl4* transcription start site drives expression in the liver but not in the intestine or islet. (A) The zebrafish *angptl4* locus and positions of promoter regions assayed in 0–7 dpf transgenic zebrafish are annotated to scale. (B) 5′ RACE and EST data (not shown) establish a single transcription start site directly upstream of exon 1. The positions of the TATA box, transcription start site, and translation start site are annotated. (C, E) Non-coding DNA −5.2 kb and −1 kb upstream of the translation start site drives expression in the liver in 6 dpf mosaic animals. Note that the −5.2 kb fragment includes a region −4.9 kb upstream from the TSS that shares extensive homology with medaka (see Figure 2A). Scale bars = 50 µm. (D, F) Liver expression pattern is confirmed in the F_1_ generation of injected animals harboring stable insertions of the −5.2 kb (*Tg(-5.2angptl4:GFP)*) and −1 kb transgenes (*Tg(-1angptl4:GFP)*). Scale bars = 50 µm. (G) Fluorescence intensity in mosaic animals is quantified (see Materials and Methods) in the liver and intestine. Circles represent mean fluorescence averaged in three mosaic patches within the liver (green) or intestine (black) of 1 fish. Note that there is minimal to no reporter expression in either the intestine or the islet (not shown). Ratios of liver or intestine positive fish versus total fish expressing GFP are shown below the corresponding construct name.
(TIF)

Figure S4 The zebrafish *angptl4* in3.4 intestinal module exhibits hallmarks of a classical enhancer. (A) *Dr* in3.4 was cloned in an inverted orientation (in3.4(ds-iv)) downstream of *GFP* driven by −1 kb of the *angptl4* promoter (*Tg(-1angptl4:GFP:in3.4inv)*). Mosaic and stable intestinal expression patterns are indistinguishable from those when in3.4 is upstream of the *Fos* minimal promoter (see Figure 3). The white arrow marks the boundary between the anterior intestine (segment 1) and mid-intestine (segment 2). The marked liver expression is likely conferred by the −1 kb *angptl4* promoter (see Figure S3F). (B) The in3.2 module drives expression of a reporter (tdTomato) in the intestinal epithelium of adult zebrafish. (C) Nuclei were isolated from adult zebrafish epithelial cells and subjected to increasing concentrations of DNase I. Digested DNA from 0.5 units DNase I was used for quantitative PCR shown in Figure 3P.
(TIF)

Figure S5 Multiple-species sequence alignment of teleost *angptl4* in3.3 modules. Sequence alignment (MUSCLE) of in3.3 regions from 12 teleost species.
(PDF)

Figure S6 Multiple-species sequence alignment of teleost *angptl4* in3.4 modules. Sequence alignment (MUSCLE) of in3.4 regions from 12 teleost species. Asterisks mark 5 individual bp changes that are differentially conserved in intestine-positive modules versus intestine-negative modules within the critical region defined by truncation mapping and SDM.
(PDF)

Figure S7 The intronic module in3.2 recapitulates microbial suppression of *angptl4*. Quantitative RT-PCR of *angptl4* and *tdT* in dissected digestive tracts from 6 dpf GF and CONVD *Tg(in3.2-Mmu.Fos:tdT)* animals. GF and CONVD animals were derived from the same *Tg(in3.2-Mmu.Fos:tdT)* stable line. *tdT* and *angptl4* mRNA were normalized to *18S* rRNA levels and are shown as fold difference compared to GF controls averaged across 3 experimental replicates ± SEM (3 biological replicate groups of 10 digestive tracts per condition per experiment). Asterisks denote P-value<.05 from unpaired T-test between GF and CONVD conditions for each gene. Note that module in3.2 includes the intestinal module in3.4 (see Figure 3).
(TIF)

Table S1 Angiopoietin-like protein sequences used for inferring phylogenic relationships.
(TXT)

Table S2 Primer sequences used in this study.
(XLS)

Table S3 Allele designations for stable lines created in this study.
(XLS)

Text S1 Text describing the comparative sequence analysis that reveals the zebrafish genome encodes a single ortholog of mammalian *Angptl4*.
(DOC)
