# Supplementary figures and images for: Intronic Cis-Regulatory Modules Mediate Tissue-Specific and Microbial Control of angptl4/fiaf Transcription
Source: PLoS Genet. 2012 Mar 29;8(3):e1002585. doi: 10.1371/journal.pgen.1002585 (PMC3315460; doi:10.1371/journal.pgen.1002585)

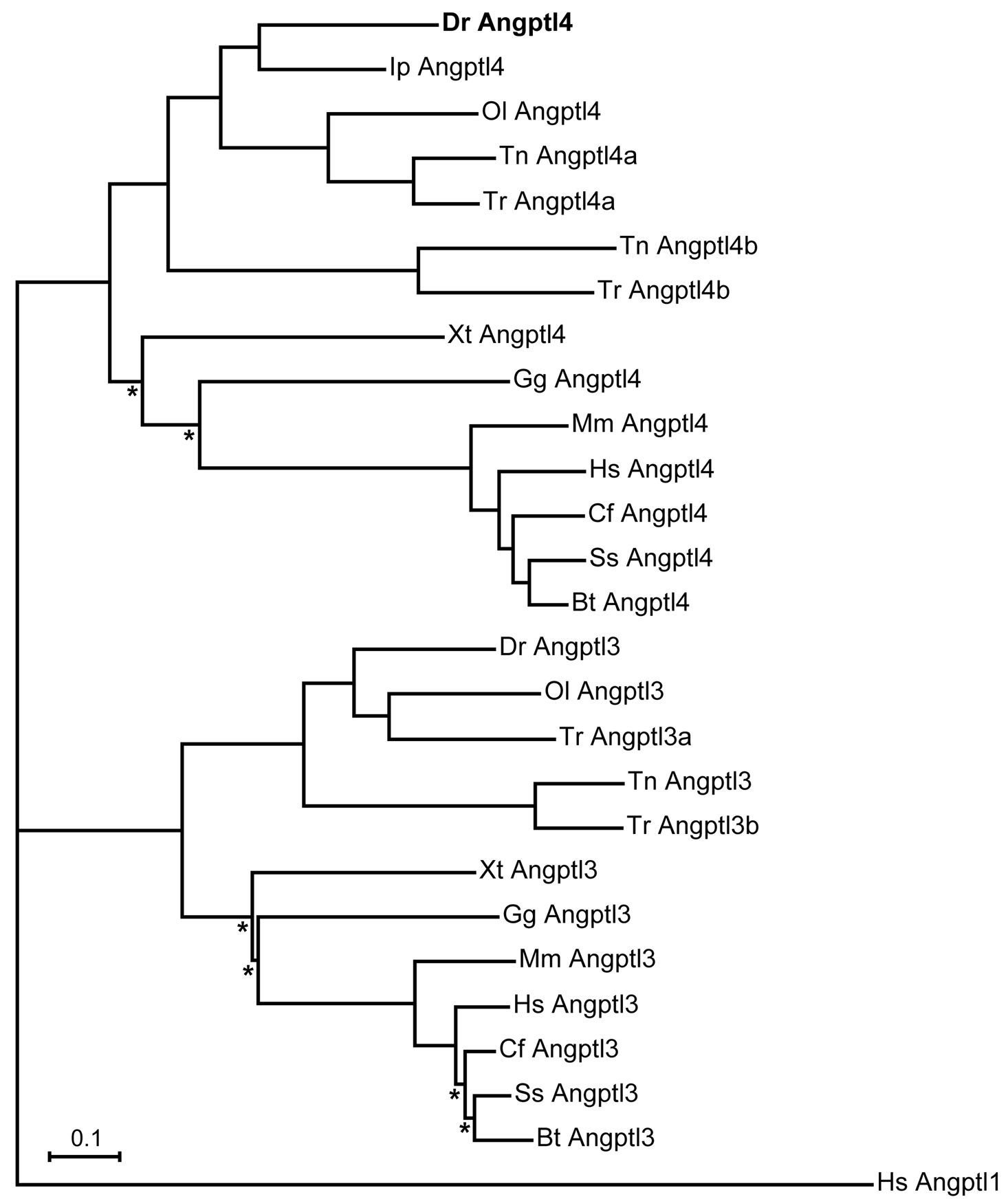

Supplement: Figure S1 — Phylogeny of Angptl4 and Angptl3 proteins from multiple vertebrate species. Distance phylogram of Angiopoietin-like 3 and 4 from zebrafish (Dr, Danio rerio), catfish (Ip, Ictalurus punctatus), medaka (Ol, Oryzias latipes), tetraodon (Tn, Tetraodoan nigroviridis), fugu (Tr, Takifugu rubipres), xenopus (Xt, Xenopus tropicalis), chicken (Gg, Gallus gallus), mouse (Mm, Mus musculus), human (Hs, Homo sapiens), dog (Cf, Canis familiaris), pig (Ss, Sus scrofa), and cow (Bt, Bos taurus). All nodes are significant (>700/1000 bootstrap replicates) except those marked with an asterisk (*). Phylogenic relationships inferred through Maximum Likelihood yield similar branching with differences only in the positions of the nodes separating Xt Angptl3 and Angptl4 and Gg Angptl3 and Angptl4 from mammals (data not shown). Scale bar indicates phylogenetic distance, in number of amino acid substitutions per site. See Table S1 for protein sequences. (TIF) [file pgen.1002585.s001.tif]

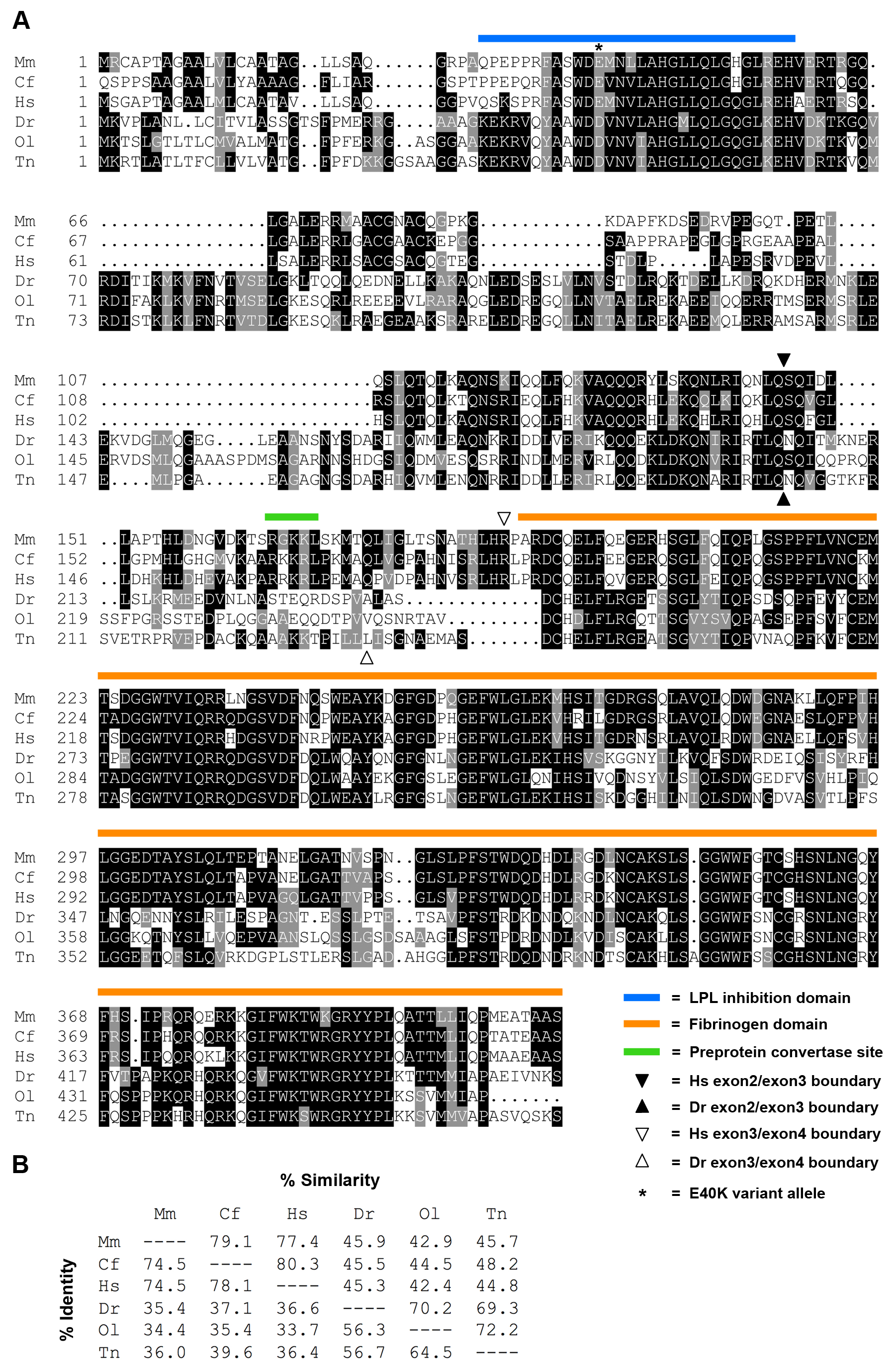

Supplement: Figure S2 — Alignment of Angptl4 proteins from multiple vertebrate species. (A) Multiple sequence alignment of Angptl4 proteins from representative vertebrate species. Amino acids highlighted in black represent identical residues in at least 50% of species, whereas amino acids highlighted in grey represent biochemically similar residues. The green line denotes the cleavage recognition sequence [91], the blue line denotes the experimentally defined LPL inhibition domain [14], and the orange line denotes the in silico predicted fibrinogen domain. Black downward arrows designate the exon 2/3 boundary in human, black upward arrows designate the exon2/3 boundary in zebrafish. White downward arrows designate the exon 3/exon 4 boundary in human, white upward arrows designate the exon 3/exon 4 boundary in zebrafish. The black asterisk marks the position of the human E40K variant [16]. (B) Percent identity and percent similarity matrix for each species pair. (TIF) [file pgen.1002585.s002.tif]

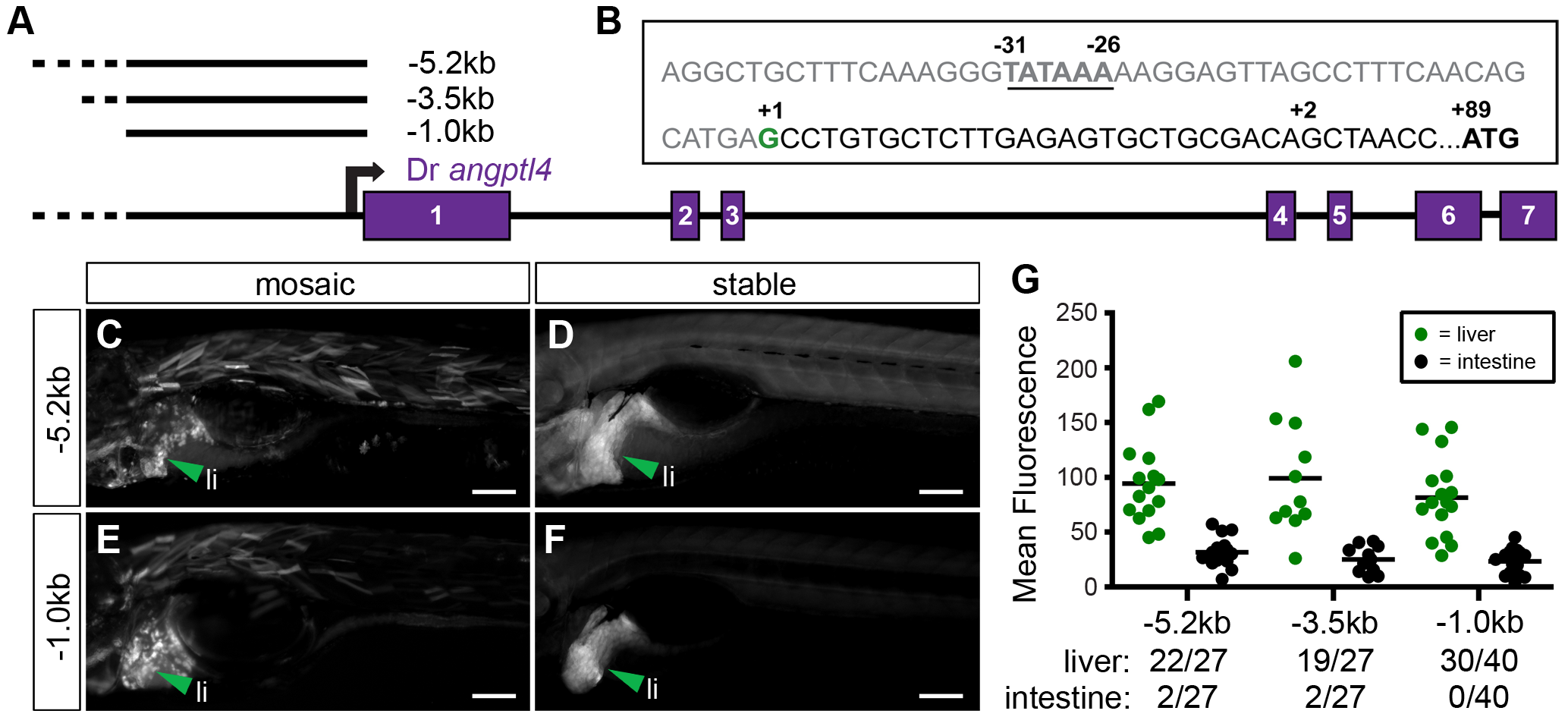

Supplement: Figure S3 — Non-coding DNA upstream of the zebrafish angptl4 transcription start site drives expression in the liver but not in the intestine or islet. (A) The zebrafish angptl4 locus and positions of promoter regions assayed in 0–7 dpf transgenic zebrafish are annotated to scale. (B) 5′ RACE and EST data (not shown) establish a single transcription start site directly upstream of exon 1. The positions of the TATA box, transcription start site, and translation start site are annotated. (C, E) Non-coding DNA −5.2 kb and −1 kb upstream of the translation start site drives expression in the liver in 6 dpf mosaic animals. Note that the −5.2 kb fragment includes a region −4.9 kb upstream from the TSS that shares extensive homology with medaka (see Figure 2A). Scale bars = 50 µm. (D, F) Liver expression pattern is confirmed in the F1 generation of injected animals harboring stable insertions of the −5.2 kb (Tg(-5.2angptl4:GFP)) and −1 kb transgenes (Tg(-1angptl4:GFP)). Scale bars = 50 µm. (G) Fluorescence intensity in mosaic animals is quantified (see Materials and Methods) in the liver and intestine. Circles represent mean fluorescence averaged in three mosaic patches within the liver (green) or intestine (black) of 1 fish. Note that there is minimal to no reporter expression in either the intestine or the islet (not shown). Ratios of liver or intestine positive fish versus total fish expressing GFP are shown below the corresponding construct name. (TIF) [file pgen.1002585.s003.tif]

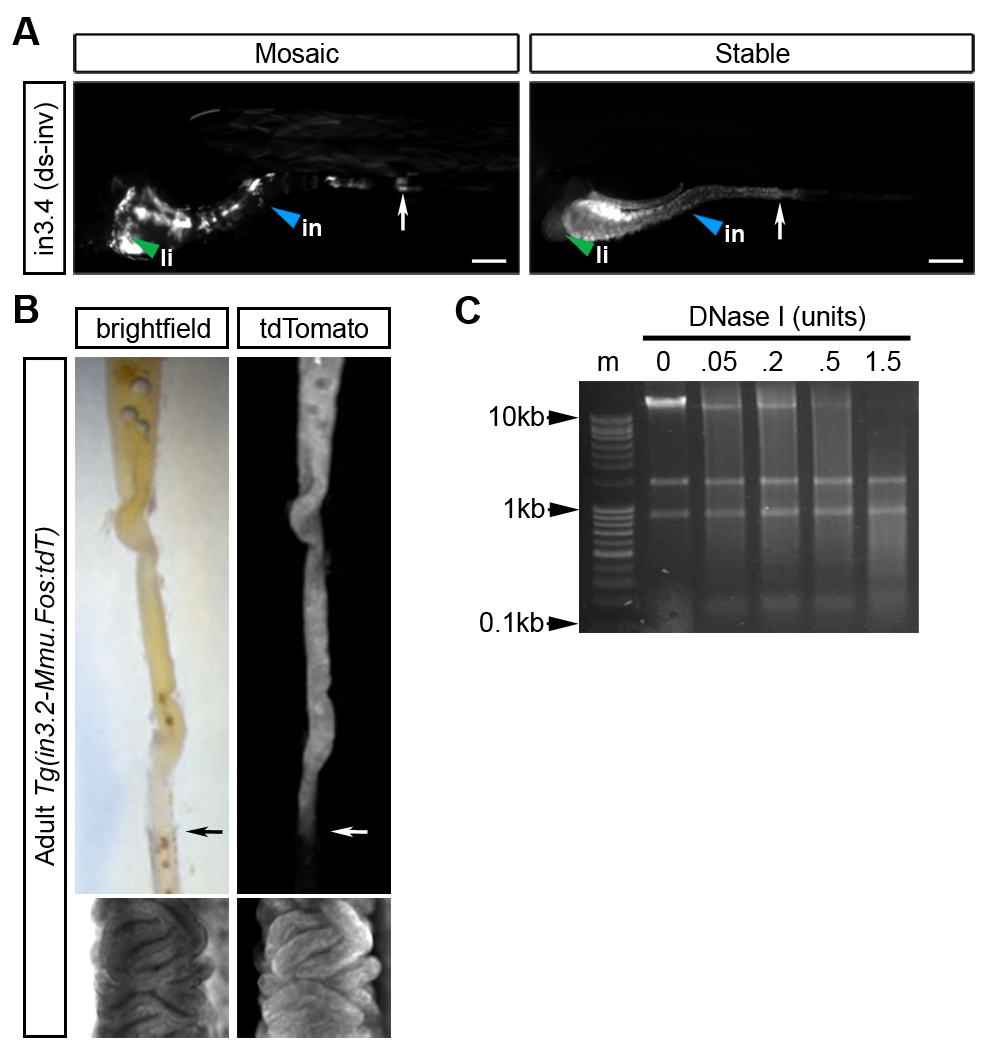

Supplement: Figure S4 — The zebrafish angptl4 in3.4 intestinal module exhibits hallmarks of a classical enhancer. (A) Dr in3.4 was cloned in an inverted orientation (in3.4(ds-iv)) downstream of GFP driven by −1 kb of the angptl4 promoter (Tg(-1angptl4:GFP:in3.4inv)). Mosaic and stable intestinal expression patterns are indistinguishable from those when in3.4 is upstream of the Fos minimal promoter (see Figure 3). The white arrow marks the boundary between the anterior intestine (segment 1) and mid-intestine (segment 2). The marked liver expression is likely conferred by the −1 kb angptl4 promoter (see Figure S3F). (B) The in3.2 module drives expression of a reporter (tdTomato) in the intestinal epithelium of adult zebrafish. (C) Nuclei were isolated from adult zebrafish epithelial cells and subjected to increasing concentrations of DNase I. Digested DNA from 0.5 units DNase I was used for quantitative PCR shown in Figure 3P. (TIF) [file pgen.1002585.s004.tif]

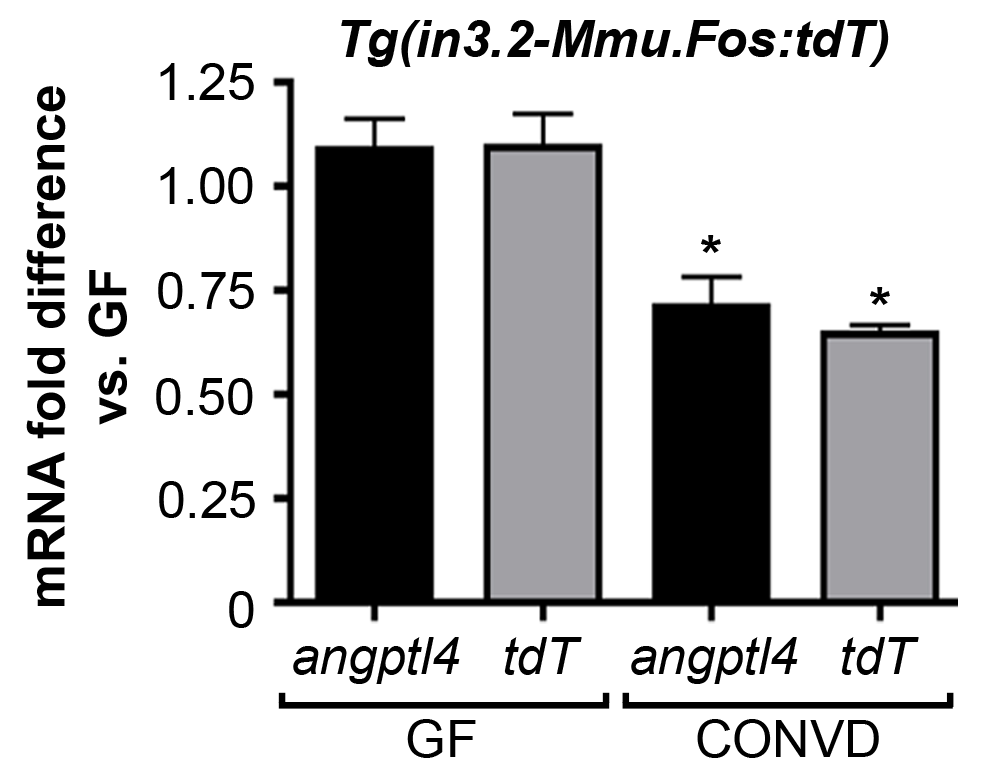

Supplement: Figure S7 — The intronic module in3.2 recapitulates microbial suppression of angptl4. Quantitative RT-PCR of angptl4 and tdT in dissected digestive tracts from 6 dpf GF and CONVD Tg(in3.2-Mmu.Fos:tdT) animals. GF and CONVD animals were derived from the same Tg(in3.2-Mmu.Fos:tdT) stable line. tdT and angptl4 mRNA were normalized to 18S rRNA levels and are shown as fold difference compared to GF controls averaged across 3 experimental replicates ± SEM (3 biological replicate groups of 10 digestive tracts per condition per experiment). Asterisks denote P-value<.05 from unpaired T-test between GF and CONVD conditions for each gene. Note that module in3.2 includes the intestinal module in3.4 (see Figure 3). (TIF) [file pgen.1002585.s007.tif]
