# Supplementary material for: Intronic Cis-Regulatory Modules Mediate Tissue-Specific and Microbial Control of angptl4/fiaf Transcription
Source: PLoS Genet. 2012 Mar 29;8(3):e1002585. doi: 10.1371/journal.pgen.1002585 (PMC3315460; doi:10.1371/journal.pgen.1002585)
Supplement: Figure S5 — Multiple-species sequence alignment of teleost angptl4 in3.3 modules. Sequence alignment (MUSCLE) of in3.3 regions from 12 teleost species. (PDF) [file pgen.1002585.s005.pdf]

```
Dr        1 GTCAG.TTAAT.........GTAGGGCATCCAA..ATTT.ATCA.GGACAGCC.ACTGCCAAAC...TTTTTATTTGGCATCTGTCTT.
Dn        1 GTCAG.TTAAT.........GTAGGGCATCCCA..ATTT.ATCA.GGACAGCC.ACTGCCAAAC...TATTTATTTGGCATCTGTCTT.
Dalb      1 GTCAG.TTAAT.........ATAGGGCATCCCA..ATTT.ATCA.GGACAGCC.ACTGCCAAAC...TTTTTATTTGGCATCTGTCTT.
Dc        1 GTCAG.TTAAT.........GTAGGGCATCCCA..ATTT.ATCA.GGACAGCC.TCTGCCAAAC...TGTTTATTTGGCATCTG...T.
Df        1 GTCAG.TTAAT.........GTAGGGCATCCCA..ATTT.AGCA.GGACAGCC.ACTGCCAAAG...TTTTTATTTGGCATCTG...T.
Daeq      1 GTCAG.TTAAT.........GTAGGGCATCCCA..ATTT.ATCACAAACAGAC.ACTGCCAAAC...TTTT..TTTGGCATCTG...T.
Ca        1 GTCAG.TTAAT.........GTAGGGCATCCAA..CTTTTAACA.GGACAGAC.AGTGCCAAA.......TTATTATAATTTTATTTTT
Cc        1 GTCAG.TTAAT.........GTAGGGCATCCAA..TTTTTAACA.GGACAGACAATTGCCAAACTTTTTTTTTTTTGGCATCTGA....
Pc        1 GTCAG.TTAAT.........GTAGGGCATCCAA..TTTT.AT............GTGCCAA.C...TTTTTTTTTGCCATCTG.....
Cm        1 AAGAGATGAAT.........ATAGGGCGCCCTG..TTTT.ATTA.GAATAT....ATAGCAAA............GAAANNTG.....
Ip        1 TTCAG.TGACTTACACAGCGAGTTGGGTGTTTAATGGTTTTAATG.GTGTAATT.GGTG.....G...TGTTTAGTAG...CCTGTTTT.
Ol        1 CTCTG.CTGAT..........GAAGGAATCCAGTAACCT.......GAGAGGC.GGCGCCG....................TGCCTG.

Dr       71 .CTCATATCCCACATGGTCCCT....TGAGGGCATGTGCATTTGCTCCTCAAATAGCACCA...GCAGCCACGGCATTCCCAGATCACTC
Dn       71 .CTCATATCCCACATAGTCCCT....TGAGGGCATGTGCATTTGCTCCTCAAATAGCAGCA...GCAGCCACGGCATTCCCGGATCACTC
Dalb     71 .CTCATATCCAACATAGCCCCT....TGAGGGCATGTGCATTTGCTCCTCAAATAGCAGCA...GCAGCCACGGCATTCCCAGATCACTC
Dc       68 .CTCATATCCCACATAGCCCCT....TGAGGGCATGTGCATTTGCTCCTTAAATAGCAACA...GCAGCCACGGCATTCCTGGATCACTC
Df       68 .CTCATATCTCACATAGTCCCT....TGAGGGCATGTGCATTTGCTCCTCAAATAGCAGCA...GCAGCCACGGCATTCCCGGATCACTC
Daeq     67 .CTCATATCCCACATAGCCCCC....TGAGGGCATGTGCAGTTGCTCCCCAAATAGCAGCG...GCAGCCTCGGCATTCCCTGATCACTC
Ca       69 ACTCCCATCTCACATAGTCCCT....TGAGGGCCTGTGCAGTTGCTTCTCAAATAGCAGCAGCAGCAGCCTCGGCATTCCCTGATCACTC
Cc       73 .CTCGCATCCCACACAGTCCCT....TGAAGGCCTGTCCAGTTGCTCCTCAAATAGCAGCA...GCAGCCTCGGCATTCCCTGATCACTC
Pc       55 .......ACTCAAATAGTCCCT....TGAGGGCCTGTGCAGTTGCTCCTCAAATAGCAGCA...GCAGTCTCGGCATTCCCAGATCACTC
Cm       55 .....CTTCCGATATAGCCCCC.....GAGGGC.TGTTTAGTTGCATCTCAAATAGCAG...........CTGCATTCCTGCATCACTC
Ip       76 ......TTCCATTGTGTCTCCT....TGAGG.........CTGCATCTCAAATGTCAGCA.........................CTC
Ol       49 .CTCCCATTCCCCGAGCTCCCCCCGCTCAGGACACCCCCATCCCCCCC......AGCATTAT..GCA.....GACACTCCTCATGCACGC

Dr      153 GCTGCCCATGCATTGTGATGTCATCAGAGGGGTGCTGTGCACGTGA.AGGAGGCGTG.....GAGAG.........CCGGACTAAAGGG
Dn      153 GCTGTCCATGCATTGTGATGTCATCAGAGGGGTGCTGTGCACGTGA.AGGAGGCGTG.....GAGAG.........CCGGACTAAAGGG
Dalb    153 GCTGTCCATGCATTGTGATGTCATCAGAGGGGTGCTGTGCACGTGA.AGGAGGCGTG.....GAGAG.........CCGGACTAAAGGG
Dc      150 GCTGTCCATGCATTGTGATGTCATGAGAGGGGTGCTGTGCACGTGA.AGGAGGCGTG.....GAGAG.........GCAGGCTAAAGGG
Df      150 GCTGTCCATGCATTGTGATGTCATCAGAGGGGTGCTGTGCACGTGA.AGGAGGCGTG.....GAGAG.........CCCGACTAAAGGG
Daeq    149 GCTGTCCATGCATTGTGATGTCATCAGAGGGGTGCTGTGCACGTGA.AGGAGGCGTG.....GAGAG.........CTGGGCTAAAGGG
Ca      155 GCTGTCCATGCATTGTGATGTCACCAGAGGGGTGCTGTGCATGTGA.AGGAGGCGTG.....GGGGG.........CTGGACTAAAGGG
Cc      155 GCTGCCCATGCACTGTGATGTCAGCAGAGGGGTGCTGTGCACGTGA.AGCAGGCGTG.....GAGGG.........CTGGACTAAAGGG
Pc      131 GCTGCCCATGCATTGTGATGTCAGCAGAGGGGTGCTGTGCACGTGA.AGCAGGCGTG.....GAAGA.........CTG..CTGGAAGG
Cm      122 GCTGCCCATGCTTTGTGAAGTCAGCAGAGGGGTGCTGTGCACGTGA.AGCAGGCGTG.....CAGGG.........CTCAATGGGTGGG
Ip      120 GCTGTTCACCCGCTGTGACGTCACACACACACTGCTGTGCACGTGA.AAGAGGCATGACTGTGTGTG.........CGCGCGTGTGTGT
Ol      125 CGCAGCCATGCGCACCAACGCCACGAGCTGGCAGCTGTGCACGTGAGGGGAGGTGCG.....GTGCGACCACCCCTCCCGCAGAAGCGGG

Dr      227 GGGCA.......GGGAGGAAAGAATGCT...........TGTA.GAGCTCTGA.........GGGAC......TGAGGAAAGTCTCTGC
Dn      227 GGGCA.......GGGAGGAAAGAATGCT...........TGTA.GAGCTCTGAGGCAACTGAGGGAC......TGAGGAAAGTCTCTGC
Dalb    227 GGGCA.......GGGAGGAAAGAATGCT...........TGTA.GAGCTCTGAG........GGANC......TGAGGAAAGTCTCTGC
Dc      224 GGGCA.......GGGAGGAAAGAATGCT...........TGAG.GAGCTCTGCGGCAACTGAGGGAC......TGAGGAAAGTCTCTGC
Df      224 GGGCA.......GGGAGGAAAGAATGCT...........TGTA.GAGCTCTGAGGCAACTGAGGGAC......TGAGGAAAGTCTCTGC
Daeq    223 GGGCA.......GGGAGTGAAGAATGCT...........AAAA.GAGCT.................C......TGAGGGAAGTCTCTGC
Ca      229 GGGCA.......GGGAGGAAGGATTGCT...........TGTA.GCTCT...............AC......TGAG...AGTCTCTGC
Cc      229 GGC.........AGGGGGAAGGGATGCT...........TGTA...GCTCT.............AC......TGAGGGATGTCTCTGC
Pc      203 GGGCA........GGGGGAAGGAATGCT...........TGTA.GCTCT...............AC......CGAGGGAAGTCTCCGC
Cm      196 TGGGATCTGGGGGGGAGGGAGGAACGTCCAGGCTCTGTAATATA.TTGCTCAGAG........GCAGC......TGAGGGAAGTCTCTGC
Ip      199 GTGCA.......TGTGTGTGCGCGTGTG...........TGTGCGCGNGCCCACGCACGTAATGAACAGCAGATGAGGGAAG..TCAGC
Ol      210 TGGAG.......GAATGTGAAGGATCCCAGAGAGCAACTCAGTA.G..TTTGGAGGTCA....GCAGC......CGCAGAGAG...CGGC

Dr      282 .TTGGGAACTGGGGGAAAGGTCATGTTT...ACACCCTCAAT.......TGTTCCAGTCAGGTCAGAATTGACATGTGAATCTGCC.TTC
Dn      291 .TTGGGAACTGGGGGAAAGGTCATGTTT...ACACCCTCAAT.......TGTTCCAGTCAGGTCAGAACTGACATGTGAATCTGCC.TTT
Dalb    283 .TTGGGAACTGGGGGAAAGGTCATGTTT...ACACCCTCAAT.......TGTTCCAGTCAGGTCAGAATTGACATGTGAATCTGCC.TTT
Dc      288 .TTGGGAACTGGGGGAAAGGTCATGTTT...ACACCCTCAAT.......TGTTCCAGTCAGGTCAGAATTGACATGTGAATCTGCC.TTT
Df      288 .TTGGGAACTAGGGGAAAGGTCATGTTT...ACACCCTCAAT.......TGTTCCAGTCAGGTCAGAATTGACATGTGAATCTGCC.TTT
Daeq    270 .TTGGGAACTGGGGGAAAGGTCATGTTT...ACACCCTCAAT.......TGTTCCAGCCAGGTCAGAATTGACATGCGAATCTGTC.TTT
Ca      274 .TTGGGAACTGGGGGAAAGGTCATGTTT...ACACCCTCAAT.......TGTTTCAGGCAGGTCAGAATTCACATGTGAAACTGCC.TTT
Cc      275 .TTGGGAACTGGGGGAAAGGTCATGTTT...ACACCCTCTAT........GTTTCAGCCAGGT..................CTGCC.TTT
Pc      250 .TTGGGAACTGGGGGAAAGGTCATGTTT...ACACCCTCAAT.......TGTTTCAGCCGGGTCAGAATTCACATGTGAAGCTGCCTTTT
Cm      271 .TTGGGAACTGGGGGAAAGGTCATGTTT...ACACCCTCAAT.......TGTTCCAGTCAGGTTACTGTTGAAATGTAAAGCTTTC.TTT
Ip      268 .NTGGGAACTAGGGGAAAGGTCATGTTT...ACACACTCATT.......GCTTCCAA................ATGGGAATCTCCAAATT
Ol      277 GCTGGGAACTGGGACAAAGGTCGTGTTTTGAACAGGAACAGTGGAGTTCTGTTCACGCTGGGTCAGA.......................

Dr      360 GGTTAGCAGATGTTTA.ACCAAA.............CACA.............................
Dn      369 GGCTGGCAGATGTTTA.GCCGAA.............TGCA.............................
Dalb    361 GTTTGGCAGATGTTAATNCCAAA.............TGGA.............................
Dc      366 GTTTGGCAGATGTTTA.GCCAAA.............TGCA.............................
Df      366 GTTTGGCAATTGCTAA.GCCAAA.............TGCA.............................
Daeq    348 GTTTGGCAGATGCTTA.GCCAAA.............TGCA.............................
Ca      352 GTTAGGCAGATGTTTG...CAAAGTGAGCCACATAGTGCA.............................
Cc      334 GTTAGGCAGATGTTN..GCCACA.............CTTGT............................
Pc      329 GTTTGGCAGATGTTC..GTCACA.............CAAG.............................
Cm      349 GTTAGGCAAATGTTTG...CAAG.............GTGATAACTCAGCTGGAAAAGAAAACAGCATTTA
Ip      331 G..CNGCAAAGG.....AACAA..............TACT.............................
Ol      344 GACGGGCCGACACTCCTTCCCTG.............AAAA.............................
```
