# Supplementary material for: Intronic Cis-Regulatory Modules Mediate Tissue-Specific and Microbial Control of angptl4/fiaf Transcription
Source: PLoS Genet. 2012 Mar 29;8(3):e1002585. doi: 10.1371/journal.pgen.1002585 (PMC3315460; doi:10.1371/journal.pgen.1002585)
Supplement: Figure S6 — Multiple-species sequence alignment of teleost angptl4 in3.4 modules. Sequence alignment (MUSCLE) of in3.4 regions from 12 teleost species. Asterisks mark 5 individual bp changes that are differentially conserved in intestine-positive modules versus intestine-negative modules within the critical region defined by truncation mapping and SDM. (PDF) [file pgen.1002585.s006.pdf]

```
Dr         1 CCTTGTAGGCTGTTGG................AAATACAAAAAT.............GC...GTGTA.GTAT..................
Dn         1 CCTTGTAGGCTGTAGC................AAATACAAAAAT.............GT...GTGTA.GTAT..................
Dalb       1 CCTTGCTGGCTGTAGC................AAATACAAAAAT.............GC...ATGTA.GTAT..................
Dc         1 CCTTGTAGGCTGTAGCATCTGGGA.......AATATACAAACAT.............GTAAAGTGTA.GTAT..................
Df         1 CCTTGTAGGCTGTAGCATCTGGGA.......AACGTACAAAAAT.............GT...GTGTA.GTAT..................
Daeq       1 CCTTGTAGTCTGTAGCATCTGGG.........AAATACTTAAAT.............GC...ATGTA.GTAC..................
Ca         1 CTTATGGTGCTTGAGGA...............ATGTATGTCAGT.............GT...GTACA.GCATTACACTGAACGTTCTTTT
Cc         1 CTGAGAA.........................ATATATGTCAGT.............GT...GTATA.GCATTACACTGAATGCTCTTTT
Pc         1 .TTTGAGAA.......................ATATATGTCAGC.............GT...GTATG.GCATTACACTGAATGCTCTTTT
Cm         1 CTGCGTATATTGTCCCATGTCTGACCAAGCTAAAATATACATATCACTTTGTGCATAGC...ATATACATAT......AAAATCTGTATT
Ip         1 GCATGTGCGCGCGCGC................ACACACACACAC.............AC...ACACACACAC..................
Ol         1 .CCTGCAGGCGG....................AGAGAGAAAAGC.............GC...............................

Dr        40 ..A...CCAACGTGGCATTTGTT........TAATAACA...ACACTTCAGTGGATTTGAACTA.TACCCTGGAGTTCAAAACAAACTCC
Dn        40 ..A...CCAACGTGGCCTTTGTT........TAATAACA......CTTCGGTGGGTTTGAACTA.TACCCTGGAGTTCAAAACAAACTCC
Dalb      40 ..A...CCAACATGGCATTTGTT........TAATAACA...ACACTTCAGTGCATTTGAACTA.TACCCTGAAGTTCAAAACAAACTCC
Dc        52 ..A...CCAATGTTGCATTTGTT........TAATAACA...ACACTTCATGTGATTTGAACTA.TACCCTGGTGTTCAAAACAAACTCC
Df        49 ..AATGCCAATGTGGCATTTGTTACACTTCATAACAACA...ACACTTCAGTGGATTTGAACTA.TACCCTAGAGTTCAAAACAAACTCC
Daeq      47 ..A...CCAATCCGGCATTTGTT........TAATAACA...ACACATCAGTTGATTCGAACTA.TACCCTGGAGATCAAAACAAACTCC
Ca        59 AGA...CCAATATGACGTTTGTT........TAATAACA...ACACGTCAGATGATTTGGACTATTTCCCTGCATTTCAAAACAAAATCC
Cc        49 GGA...CCAGTGTGAGATTTGTT........TAATAACA...ACTCATCAGATGACTTGGACTATTGCCCTGGAGTTCAAAACAAACTCC
Pc        50 GCA...CCAGTGTGAGATTTGTT........TAATAACA...ACATGGCAGATGACTTGGACTATTGGCCCTGAGTTCAAAACAACCTCC
Cm        82 GAA...CAAATATGGTATT............TAAGGACA...ACATGTCAAAAGATTTGGACTATTGCCCTGGAATTCAAAACAAG....
Ip        41 ..A...CACACACA.................CACACACACACACACACACACAGATTTTAATTTGTCCAGTTAACCTAAATAAAAATCTC
Ol        26 ..A..........GGTTTCCTTG........TAAAAAAA.....GCTTCTCCGAGTC....CTGCGGCCTCGATCTTCA.........TC
                                       *          *           *               *        *
Dr       113 ATGCTGGC........TTCTGGGTTCTTGGG.TGACATGTTCAAGGTCCAGTGTTTGAGATAAGGATTT..AGTACACCATTAAACAATA
Dn       110 ATGCTGGC........TTCTGGGTTCTTGGG.TGACATGTTCAAGGTCCAGTGTTTGAGATAAGGATTT..AGTACACCATTAAACAATG
Dalb     113 ATGCTGGC........TTCTGGGTTCTTGGG.TGACATGTTCAAAGTCCAGTGTTTGAGATAAGGATTTTANGTACACCATTAAACAATG
Dc       125 ATGCTGGC........TTCTGGGTTCTTGGG.TGACATGTTCAAGGTCCAGTGTTTGAGATAAGGATTT..AGTACACCATTAAACAATG
Df       133 ATGCTGGC........TTCTGGGTTCTTGGG.TGACATGTTCAAGGTCCAGTGTTTGAGATAAGGATTT..AGTACACCATTAAACAATG
Daeq     120 ATGCTGGC........TTCTGGGTTCCTGGG.TGACACGTTCAAGGTCCCGTGTTTGAGATAAGGCTTT..AGTTCACCATTAAACAATG
Ca       135 ATGCTGAC........TTTTGGATTCTTGGG.TGACACTGATAAGATTCTGTCTTTGAGATAAGGTTT...AGTTCACCATTAAGCAATG
Cc       125 ATGTTGGC........TTTCGGGTTCTTGGA.TGGCACGTTCAAGGTTCTGTGTTTGAGATAAAGTTT...AGTTCACCATTAAACAATG
Pc       126 ATGTTGGC........TTTTGGGTTCTTGGGTTGGCACGTTCAAGGTTCTGCAGTTGAGATAAGGTTT...AGTTCACCATGT.......
Cm       150 AAGTTGGT........TTCTGGGTACT.............TCAAGGTTCTGTGACTGCGATAAGGATTT..AGTTTGCTATTAAACAATG
Ip       109 ACACT...........................TAAGATNTTTAC.............ATATAAG.......CGTACACACACACACACA.
Ol        78 ACTTTGGCAGAAACATTTTCAGGTTTTCAGACTGATAT....AAAACCCAGCGG.........................GTCACACGCTG

Dr       192 A.GATAAACACTTATCCTGGACGTGTGAGCGTTTTAAATACT.TTGGCAACTTTAACATCTTTGTTTGGGTAC..AGCCTTGGGCAAAGG
Dn       189 A.GATAAACCATTAT............................TTGCCAACTTTAAAATCTCTGTTTGGGTAC..AGGTTTGGGCAAAGG
Dalb     194 A.GATAAACCCTTATCCTGGACGTGTGAGCGATTTCCATTCT.TTACCAACTTTAAAATGTCTGTTTGGGTAC..AGGCTTGGGCAAAGG
Dc       204 A.GATAAACGCTTATCCTGAACGTGTGAGCGATTTCCATTCT.TTGCCAACTTTAAAATCTCTGTTTGGGTAC..AGGCTTGGGCAAAGG
Df       212 A.GATAAAGGCTTATCCTGGACGTGTGAGCGGTTTCCATTTT.TTGCCAACTTTAAAATCTCTGTTTGGCTAC..AGGCTTGGGCAAAGG
Daeq     199 ..............................................CCAACTTTAAAATCTCTGTTTGGGTAC..AAGCTTGGACAAAGG
Ca       213 GAGATAAACACTTGTTCTGAATGTGTGAGCAATTTCCATTTA.GTGCCAACTTTAACATCCCTGTTTGTGGAT..AGGCTTAGGGAGAGG
Cc       203 T.GATAAACCTTTACCCTAGACATGTGAACAATTTCCATTTA.GTGCCAATTTATAAATCCCTGTTTGGATAC..AGGCTTGGGTAAAGG
Pc       198 ..GATAAACTTTTACCCTAGACGTGCTAGCGGTTTCCATTTTACTGCCAACTTTGGCAT....GTTTGTATAC..AGGCTTGAGTAAAGG
Cm       217 A.GATGAACAC.............ATGAGAAGTTTCCAGTCT.GTGCCAACTATGGAATCCCTATTAGAATACTTATGACTG...ACAAG
Ip       151 ..CACACACACCTATCT..........................GTGCTG....................................AAATG
Ol       139 GAGTCAAACCCCAA.............................TTCCCAAGGTTAACCT................GGGATTGAGGGGATC

Dr       278 TCATTTGCAGATGCTTGAACA.........TGTGTTTG....TGTCTTTCAG
Dn       248 TCATTTGCAAATGCTTGAACA.........TGTGTTTG....TGTCTTTCAG
Dalb     280 TCACTTGCAGATGCTTGAACA.........TGCGTTTG....TGTCTTTCAG
Dc       290 TCATTTGCAGATGCTTGAACG.........TGTGTTTG....TGTCTTTCAG
Df       298 TCATTTGCAGATGCTTGAACA.........TGTGTTTG....TGTCTTTCAG
Daeq     241 TCATTTGCAGATGCTTGAACA.........TGTGTTTG....TGTCTTTCAG
Ca       300 TAATTTGCAGATGTGTGAACA..TTTGTGGTGTTTTTG....TATCTTTCAG
Cc       289 TCATTTGCAGACATGTGAACATTTTTGTGGTGTTTTGG....TGTCTTTCAG
Pc       280 TCATTTGCAGACGTGTGAACATTTTTGTGGTGTTTTGT....TGTCTTTCAG
Cm       289 TGACATGTAGATATGTAAACA..TGTATGTTTTGTTTT....TGTTTTACAG
Ip       177 TTATGT.........TAAACG.........TGTGTTTAATGTTATTTTGCA.
Ol       184 CAGACAGCAGCAGCGTGTACT.......CATCCGCCCTAAAATGTCTTTCAG
```
