# Supplementary material for: Intronic Cis-Regulatory Modules Mediate Tissue-Specific and Microbial Control of angptl4/fiaf Transcription
Source: PLoS Genet. 2012 Mar 29;8(3):e1002585. doi: 10.1371/journal.pgen.1002585 (PMC3315460; doi:10.1371/journal.pgen.1002585)
Supplement: Text S1 — Text describing the comparative sequence analysis that reveals the zebrafish genome encodes a single ortholog of mammalian Angptl4. (DOC) [file pgen.1002585.s011.doc]

**Text S1: The zebrafish genome encodes a single ortholog of mammalian *Angptl4***

We conducted a comparative genome sequence analysis to determine how the partial genome duplications that occurred during teleost evolution [1,2] affected *Angptl4* gene number in different teleost lineages. We queried vertebrate genome, cDNA, and EST databases for Angiopoietin-like proteins and inferred phylogenic relationships between Angiopoietin-like family members. We found that the genomes of zebrafish, channel catfish (*Ictaluris punctatus*), and medaka (*Oryzias latipes*) encode a single ortholog of mammalian Angptl4, whereas two pufferfish species (*Takifugu rubripes* and *Tetraodon nigroviridis*) encode two Angptl4 paralogs (Figure 1A and Figure S1). Alignment of Angptl4 protein sequences from zebrafish, medaka, human (*Homo sapiens*), and mouse (*Mus musculus*) revealed strong amino acid sequence conservation between fishes and mammals (Figure S2). Sequence conservation was highest in the C-terminal fibrinogen domain and a N-terminal domain involved in binding and inhibiting LPL [3,4]. These results indicate that the zebrafish genome encodes a single ortholog of mammalian *Angptl4*, which exhibits strong evolutionary conservation in domains comprising the known functional capacity of this protein.

REFERENCES

**1. Amores A, Force A, Yan YL, Joly L, Amemiya C, et al. (1998) Zebrafish hox clusters and vertebrate genome evolution. Science 282: 1711-1714.**

**2. Kasahara M, Naruse K, Sasaki S, Nakatani Y, Qu W, et al. (2007) The medaka draft genome and insights into vertebrate genome evolution. Nature 447: 714-719.**

**3. Lee EC, Desai U, Gololobov G, Hong S, Feng X, et al. (2009) Identification of a new functional domain in angiopoietin-like 3 (ANGPTL3) and angiopoietin-like 4 (ANGPTL4) involved in binding and inhibition of lipoprotein lipase (LPL). J Biol Chem 284: 13735-13745.**

**4. Yau MH, Wang Y, Lam KS, Zhang J, Wu D, et al. (2009) A highly conserved motif within the NH2-terminal coiled-coil domain of angiopoietin-like protein 4 confers its inhibitory effects on lipoprotein lipase by disrupting the enzyme dimerization. J Biol Chem 284: 11942-11952.**
